# BV-2 Microglial Cells Overexpressing *C9orf72* Hexanucleotide Repeat Expansion Produce DPR Proteins and Show Normal Functionality but No RNA Foci

**DOI:** 10.3389/fneur.2020.550140

**Published:** 2020-10-06

**Authors:** Hannah Rostalski, Tomi Hietanen, Stina Leskelä, Andrea Behánová, Ali Abdollahzadeh, Rebekka Wittrahm, Petra Mäkinen, Nadine Huber, Dorit Hoffmann, Eino Solje, Anne M. Remes, Teemu Natunen, Mari Takalo, Jussi Tohka, Mikko Hiltunen, Annakaisa Haapasalo

**Affiliations:** ^1^A.I. Virtanen Institute for Molecular Sciences, University of Eastern Finland, Kuopio, Finland; ^2^Institute of Biomedicine, University of Eastern Finland, Kuopio, Finland; ^3^Institute of Clinical Medicine—Neurology, University of Eastern Finland, Kuopio, Finland; ^4^Neuro Center, Neurology, Kuopio University Hospital, Kuopio, Finland; ^5^Unit of Clinical Neuroscience, Neurology, University of Oulu, Oulu, Finland; ^6^Medical Research Center (MRC) Oulu, Oulu University Hospital, Oulu, Finland

**Keywords:** amyotrophic lateral sclerosis, BV-2 cells, *C9orf72* DPRs, *C9orf72* hexanucleotide repeat expansion, frontotemporal lobar degeneration, microglia, neuroinflammation, TDP-43

## Abstract

Hexanucleotide repeat expansion (HRE) in the *chromosome 9 open-reading frame 72* (*C9orf72*) gene is the most common genetic cause underpinning frontotemporal lobar degeneration (FTLD) and amyotrophic lateral sclerosis (ALS). It leads to the accumulation of toxic RNA foci and various dipeptide repeat (DPR) proteins into cells. These *C9orf72* HRE-specific hallmarks are abundant in neurons. So far, the role of microglia, the immune cells of the brain, in *C9orf72* HRE-associated FTLD/ALS is unclear. In this study, we overexpressed *C9orf72* HRE of a pathological length in the BV-2 microglial cell line and used biochemical methods and fluorescence imaging to investigate its effects on their phenotype, viability, and functionality. We found that BV-2 cells expressing the *C9orf72* HRE presented strong expression of specific DPR proteins but no sense RNA foci. Transiently increased levels of cytoplasmic TAR DNA-binding protein 43 (TDP-43), slightly altered levels of p62 and lysosome-associated membrane protein (LAMP) 2A, and reduced levels of polyubiquitinylated proteins, but no signs of cell death were detected in HRE overexpressing cells. Overexpression of the *C9orf72* HRE did not affect BV-2 cell phagocytic activity or response to an inflammatory stimulus, nor did it shift their RNA profile toward disease-associated microglia. These findings suggest that DPR proteins do not affect microglial cell viability or functionality in BV-2 cells. However, additional studies in other models are required to further elucidate the role of *C9orf72* HRE in microglia.

## Introduction

Microglia are resident immune cells in the brain that perform vital functions during brain development, homeostasis, and aging. These include migration, phagocytosis of cell debris, pathogens, or excess or non-functional synapses, as well as sensing environmental stimuli and switching their phenotype and function accordingly (1). Defects in microglial function and chronic changes in their physiology have been associated with a variety of developmental and neurodegenerative diseases, but the exact role of microglia in the pathogenesis of frontotemporal lobar degeneration (FTLD) and amyotrophic lateral sclerosis (ALS) is not known (2–5).

FTLD and ALS are neurodegenerative disorders within the same disease spectrum and with overlapping pathological features and genetic background. However, the clinical phenotypes and the pattern of atrophy of these diseases differ remarkably (6–10). A *GGGGCC* hexanucleotide repeat expansion (HRE) in the intronic region of the *chromosome 9 open-reading frame 72* (*C9orf72*) gene is the most common genetic cause underpinning both FTLD, ALS, and combined FTLD-ALS (11–14). This repeat expansion leads to reduced mRNA and protein levels of all human C9orf72 isoforms (C9orf72 haploinsufficiency), suggesting a loss-of-function mechanism (15). Furthermore, RNA-binding proteins (RBPs) bind to the expanded repeat-containing RNA, forming RNA foci in the nucleus and sometimes in the cytosol (16–22). The sequestration of the RBPs and inhibition of their normal function might contribute to neuronal loss (18, 23). The expanded repeat-containing RNA can also be translated into dipeptide repeat (DPR) proteins via repeat-associated non-AUG translation. Because the transcription of the expanded repeat sequence takes place in both sense and antisense direction, five different DPR protein species can be generated (poly-GA, poly-GR, poly-GP, poly-PA, poly-PR) (15, 24–29). These DPR proteins can lead to cell toxicity through different pathways such as perturbation in RNA processing, proteasomal inhibition, and induction of apoptosis (30–34). Formation of the RNA foci and generation of the DPR proteins represent the gain-of-toxic-function mechanisms involved in the *C9orf72* HRE-associated disease pathogenesis. In addition to these specific pathological hallmarks present only in the *C9orf72* HRE carriers, inclusions of accumulated Sequestosome 1/p62 and TAR DNA-binding protein (TDP)-43 have been detected in FTLD and ALS patients, including *C9orf72* HRE carriers (35–41). *C9orf72* HRE-derived pathological hallmarks and their potential downstream effects have been mostly described in neuronal cells, but so far, only a few studies have included other cell types, such as glial cells (16, 17, 20, 21, 42). Because glial cells have been pinpointed as potential contributors to neurodegenerative diseases, elucidating their role in *C9orf72* HRE-associated FTLD and ALS is necessary.

Here, we have investigated the effects of the *C9orf72* HRE on microglial cells by introducing the *C9orf72* HRE into mouse BV-2 cells and assessing the presence of the *C9orf72* HRE-associated pathological hallmarks and microglial cell functionality. Our results suggest that microglial cells harboring the *C9orf72* HRE present specific HRE-associated pathological hallmarks but remain functional.

## Materials and Methods

### BV-2 and N2a Cell Cultures

Mouse BV-2 cells (43) were cultured in RPMI-1640 medium (R7509, Sigma-Aldrich) supplemented with 2.4 mM L-glutamine (17-605E; Gibco), 10% (*v*/*v*) fetal bovine serum (10270-106; Gibco), and 1.2% (*v*/*v*) penicillin/streptomycin (15140-122; Gibco). BV-2 cells were scraped off gently from cell culture plates (168381; Thermo Scientific) and collected by centrifugation at room temperature (RT) for 3 min at 800 × *g*. Cell pellets were resuspended in cell culture medium and plated for maintenance or experiments on cell culture plates or poly-l-lysine (P6282; Sigma-Aldrich) coated glass coverslips. Mouse Neuro-2a (N2a) neuroblastoma cells (ATCC CCL-131; ATCC) were maintained in Dulbecco's Modified Eagle's Medium (BE12-614F; Lonza) containing 2.4 mM L-glutamine, 10% (*v*/*v*) fetal bovine serum, and 1.2% (*v*/*v*) penicillin/streptomycin. N2a cells were washed once with warm Dulbecco's phosphate-buffered saline (DPBS, 17-512F; Lonza) and incubated in 0.25% trypsin solution (in DPBS, 15090-046; Gibco) for 3–5 min at +37°C. Next, the cell suspension was diluted in the culture medium and centrifuged for 3 min at 800 × *g*. The supernatant was discarded, and the cells were resuspended in the culture medium and plated for experiments on poly-d-lysine (P6407, Sigma-Aldrich) coated coverslips or on cell culture plates for maintenance.

### Transfections and Treatments

Control (2R) and *GGGGCC* hexanucleotide repeat expansion-containing plasmid (66R) (44) were maxiprepped using NEB Stable Component *E. coli* (C3040H, New England Biolabs) and purified using QIAfilter Plasmid Maxi Kit (12262, Qiagen). BV-2 cells were transfected with either 2R or 66R plasmids using Magnetofection (GL00250; OZ Biosciences) according to manufacturers' instructions. For cell viability assay and to transfect N2a cells, Viromer Yellow transfection reagent (VY-01LB-01; Lipocalyx, Halle, Germany) was used according to the instructions provided by the manufacturer. For microscopy-based approaches, 2R or 66R plasmids were used in combination with a pLVX-IRES-ZsGreen1 vector (pLVX plasmid, 632187; Clontech Laboratories) to detect transfected cells based on *Zoanthus* sp. green fluorescent protein (ZsGreen) 1 fluorescence. In some experiments, BV-2 cells were treated for 24 h with 200 ng/mL lipopolysaccharide (L5543; Sigma-Aldrich) and 20 ng/mL interferon-γ (14777; Sigma-Aldrich) in DPBS. Cells treated with equal volumes of DPBS were used as vehicle controls.

### Protein Extraction and Western Blotting

Twenty-four or 48 h after transfection, cells were washed twice with cold DPBS (D8537; Sigma-Aldrich) and scraped in lysis buffer (10 mM Tris–HCl, 2 mM EDTA, 1% SDS) supplemented with 1:100 protease and 1:100 phosphatase inhibitors (1862209 and 1862495; Thermo Scientific). Before protein concentration measurement, samples were sonicated (2 cycles, each cycle 10 s, 30 s between cycles, high setting; Bioruptor Next Gen, Diagenode) and boiled at +85°C for 7 min. Protein concentrations were measured using bicinchoninic acid assay (23225; Thermo Scientific) and plate reader (Infinite M200; Tecan Group Ltd.). Samples and molecular weight marker (26616; Thermo Scientific) were supplied with 20% (*v*/*v*) 2-mercaptoethanol and 1× NuPAGE LDS sample buffer (NP0007; Invitrogen), heated for 7 min at +85°C, and loaded on 4–12% Bis-Tris gels (NP0335; Invitrogen) for sodium dodecyl sulfate–polyacrylamide gel electrophoresis (80 V for 15 min, then 110 V for 1 h 15 min). Proteins were transferred on 0.2 μm polyvinylidene fluoride membranes (1704157; Bio-Rad) using Trans-Blot Turbo Transfer System (Bio-Rad, 25 V, 1.0 A, 30 min). Blocking was done with either 5% (*w*/*v*) milk powder or 5% (*w*/*v*) BSA (A9647; Sigma-Aldrich) diluted in 1× TBST (139.97 mM NaCl, 24.94 mM Tris, 0.5% (*v*/*v*) Tween 20; pH 7.4) for 1 h at RT according to the recommendations of primary antibody manufacturers. Blots were incubated overnight at +4°C with the following primary antibodies diluted in 1× TBST: anti-C9orf72 (1:500, 22637-1-AP; Proteintech), anti-caspase 3 (1:1,000, 9662; Cell Signaling Technology), anti-poly-GA (1:10,000, MABN889; EMD Millipore), anti-poly-GP (1:2,000, ABN455; EMD Millipore), anti-poly-GR (1:5,000, MABN778; EMD Millipore), anti-LAMP2A (1:1,000, ab18528; Abcam), anti-LC3B (1:3,000, ab51520; Abcam), anti-phospho-TDP-43 (1:1,500, TIP-PTD-P02; CosmoBio), anti-TDP-43 (1:1,000, 10782-2-AP; Proteintech), anti-p62 (1:1,000, 51142; CST), anti-polyubiquitinylated proteins (FK1, 1:1,000, BML-PW8805-0500; Enzo Life Sciences), anti-Hsp70 (1:5,000, ADI-SPA-810; Enzo Life Sciences), and anti-β-actin (1:1,000, ab8226; Abcam). Afterward, the blots were incubated with species-specific horseradish peroxidase-linked secondary antibodies (1:5,000, NA935, NA934V, or NA931V; GE Healthcare) for 1 h at RT, and for 5 min with suitable enhanced chemiluminescence substrate solutions (RPN2236 or RPN2235; GE Healthcare). The chemiluminescence signal was detected using ChemiDoc MP Imaging System (Bio-Rad). For each sample, bands of interest were quantified using Image Lab (6.0.0; Bio-Rad) and normalized to the β-actin signal in the same samples. Data are shown as percentage of 2R (mean value of each experiment set to 100%).

### Fluorescence *in situ* Hybridization (FISH) and Immunocytochemistry

Cells were fixed with 4% (*v*/*v*) formaldehyde (28908; Thermo Scientific) in DPBS for 20 min at RT, washed two times with DPBS, and stored at +4°C. FISH was performed as described in Chew et al. (44) using fluorescently labeled locked nucleic acids, TYE 563-(CCCCGG)_3_, to detect the sense foci. TYE 563-(CAG)_6_ was used as a negative control probe (Exiqon). For immunocytochemistry, cells were permeabilized using 0.1% (*v*/*v*) Triton X-100 in DPBS for 10 min at RT on a rocker and washed three times with DPBS. For blocking, cells were incubated in blocking solution (0.015% (*v*/*v*) goat immunoglobulin G isotype control (02-6202; Invitrogen) in DPBS) for 30–50 min at RT on a rocker. Primary and secondary antibodies were diluted in blocking solution and incubated with the cells either at RT for 1.5 h or overnight at +4°C followed by washing three times with DPBS. Immunocytochemistry was performed using the following primary antibodies: anti-poly-GA (1:500, MABN889; EMD Millipore), anti-poly-GP (1:500, ABN455; EMD Millipore), anti-poly-GR (1:500, MABN778; EMD Millipore), anti-p62 (1:200, sc-48402; Santa Cruz Biotechnology), anti-LAMP2A (1:200, ab18528; Abcam), and anti-TDP-43 (1:500, 10782-2-AP; Proteintech). The following fluorescently labeled species-specific secondary antibodies were used in 1:500 dilutions: Alexa Fluor 568 (A-11004; Invitrogen), Alexa Fluor 594 (A-11007; Invitrogen), and Alexa Fluor 647 (A-21244; Invitrogen). To stain the cell nuclei, cells were either incubated with 4′,6-diamidino-2-phenylindole (DAPI, D9542; Sigma-Aldrich) 1:5,000 in DPBS for 5 min at RT on a rocker and washed three times with DPBS and mounted (345789; Calbiochem) or directly mounted in DAPI-containing mounting medium (H-1800; Vector Laboratories). Confocal images were acquired with LSM800 confocal microscope (Zeiss). In independent experiments, sample and control slides were imaged with the same microscopy settings.

### RNA Extraction, cDNA Synthesis, and RT-qPCR

BV-2 cells were transfected with either 2R or 66R plasmid as described previously. Twenty-four or 48 h after transfection, cells were washed twice with ice-cold DPBS and scraped in 200 μL ice-cold DPBS. Samples were stored at −80°C until total RNA was extracted (11828665001; Roche Molecular Systems). RNA concentrations were measured using NanoDrop One (Thermo Scientific). Per sample, 1 μg of RNA was reverse transcribed into cDNA using random hexamer primers (04897030001; Roche Molecular Systems). Real-time quantitative PCR was performed using SYBR Green I (04707516001; Roche Molecular Systems) and LightCycler 480 II (Roche Molecular Systems).

Primer pairs and annealing temperatures are listed in Supplementary Table 1. Samples were run in duplicates. Thermal cycler conditions were set as follows: 5 min +95°C pre-incubation followed by 45 cycles of 1) 10 s +95°C denaturation, 2) 15 s annealing, and 3) 20 s +72°C elongation. Expression values of genes of interest were calculated using the ΔΔC_T_ method and normalized to expression levels of β*-actin*. For each time point and experiment, mean expression values of 2R samples were set to 100%. Expression values of 66R samples were normalized to mean expression values of 2R samples for each experiment separately. Amplification specificity was checked using melting curves (LightCycler 480 software 1.5.1.62 SP3; Roche Molecular Systems). All the primer pairs used here gave only one peak in the melting curves.

### TDP-43 Translocation Analysis

BV-2 cells were co-transfected with either 2R or 66R plasmids in combination with pLVX plasmid, fixed 24 or 48 h after transfection, and double-stained with anti-poly-GA (and goat anti-mouse Alexa Fluor 568) and anti-TDP-43 (and goat anti-rabbit Alexa Fluor 647) antibodies as described previously. Confocal microscopy images were acquired from three (24 h) and two (48 h) independent cell culture experiments using laser-scanning microscopy (LSM800; Zeiss). An automated segmentation technique was developed in MATLAB to annotate cell nuclei and cell bodies in images. The images included four channels: AF647 (for goat anti-rabbit Alexa Fluor 647), AF568 (for goat anti-mouse Alexa Fluor 568), ZsGreen1, and DAPI. The ZsGreen1 and DAPI channels marked cell bodies and cell nuclei, respectively. An iterative 2D median filtering, 10 iterations with a window size of 7 × 7 pixels, was applied on the images of ZsGreen1 and DAPI channels to smooth cell bodies and cell nuclei for the segmentation. A preliminary segmentation of cell nuclei was acquired by thresholding the DAPI channel for intensity values above 20, image intensities ranged from 0 to 255, and subsequently, the connected component analysis was applied. Components with an area smaller than 5,000 pixels (21.125 μm^2^) were discarded. Holes in segmented components were filled using morphological operations. The preliminary segmentation of cell nuclei contained under-segmentation errors, which were eliminated using the watershed transform. The location of markers was determined using the regional minima of the Euclidean distance transform of the preliminary segmentation of cell nuclei. The same procedure was applied to segment cell bodies from the ZsGreen1 channel. The threshold, at which the preliminary segmentation of cell bodies was acquired, was set to 1 because only the background was equal to zero in this channel. Components of an area smaller than 3,000 pixels (12.675 μm^2^) were discarded, and holes were filled using morphological operations. The individual cell nuclei were assigned as markers for a marker-based watershed segmentation, addressing under-segmentation error in cell bodies. Cells with cell body average intensity value below 6 were eliminated from the final segmentation. This threshold was set based on the average intensity of all cell bodies. In addition, apoptotic cells were eliminated from the segmentation. Apoptotic cells were defined as cells in which the spatial distribution of the intensity values of the cell body is heterogeneous, i.e., a concentration of high-intensity values within the cell body of an area smaller than the nucleus. For each cell, the final segmentation for the area of cell body, the area of a nucleus, the sum intensities of cell body in AF647 channel, and the sum of the intensities of a nucleus in the AF647 channel were quantified. An exemplary picture of the segmentation toolbox is shown in Supplementary Figure 1. The source code is freely available at https://github.com/AndreaBehan/FMIS-software.

For TDP-43 translocation analysis, four stages of intracellular TDP-43 distribution were defined depending on the ratio of nuclear to cytoplasmic TDP-43 signal (see **Figure 4**): none (TDP-43 signal—if any—is only present in the nucleus, and cytoplasmic signal is below threshold of cytoplasmic background control); mild (cytoplasmic TDP-43 signal is above the threshold of the cytoplasmic background control but below nuclear TDP-43 signal, which is above the threshold of the nuclear background control); moderate (nuclear TDP-43 signal is above the threshold of nuclear background control, and the cytoplasmic TDP-43 signal, which is above the threshold of the cytoplasmic background control signal, is above the nuclear TDP-43 signal); severe (nuclear TDP-43 signal is below the threshold of nuclear background control, and the cytoplasmic TDP-43 signal is above the threshold of the cytoplasmic background control). TDP-43 signal intensities were measured as SUM intensities of AF647 channel within the nucleus or cytoplasm (nuclear signal subtracted from whole cell-signal) as described previously and normalized to the nuclear or cytoplasmic area. Nuclear and cytoplasmic background control values were obtained by calculating AF647 SUM intensity in the nucleus (DAPI positive area) and cytoplasm (ZsGreen1 positive, DAPI negative area) of ZsGreen1-positive 2R or 66R cells immunostained without primary antibody. Cells were excluded from the analysis if located on edges of the images or if cell compartments were not well-recognized by the program.

### p62 and LAMP2A Single-Cell Analysis

BV-2 cells were co-transfected with either 2R or 66R plasmids in combination with pLVX plasmid, fixed 24 or 48 h after transfection, and stained with anti-p62 or anti-LAMP2A antibodies followed by goat anti-mouse Alexa Fluor 568. Images were acquired from three (LAMP2A) and two (p62) independent cell culture experiments using laser-scanning confocal microscopy (LSM800; Zeiss). Fluorescence intensities of Alexa Fluor 568 were quantified in ZsGreen1-positive cells using ImageJ (Rasband, W.S., ImageJ, U. S. National Institutes of Health, Bethesda, Maryland, USA, https://imagej.nih.gov/ij/, 1997-2018). SUM intensity values were normalized to cell areas. For each experiment, average area-normalized SUM intensity values of negative control pictures (cells stained without primary antibody) were subtracted from area-normalized SUM intensity values of samples.

### Nitrite Assay and TNFα, IL-1β, and IL-6 ELISA

BV-2 cells were treated 24 h post-transfection with LPS/IFNγ as described previously. After the 24-h treatment, the culture medium was collected, centrifuged at 10,000 × *g* for 10 min at +4°C, and supernatants were stored at −20°C until analysis. Nitrite levels (as an indication of nitric oxide production) of three technical replicates per each biological replicate were measured via Griess reaction (G-7921; Invitrogen). To calculate the nitrite concentration for each biological replicate, means of their corresponding technical replicates were used. Extracellular tumor necrosis factor alpha (TNFα) (88-7324-22; Invitrogen), interleukin (IL)-1β (88-7013-22; Invitrogen), and IL-6 (88-7064-22; Invitrogen) concentrations were measured using ELISA according to kit instructions. For ELISA, no technical replicates were used. Absorbance was measured using a plate reader (Infinite M200, Tecan Group Ltd.). Data are shown as concentrations obtained from the standard curve. Data were obtained from three independent experiments, each including three biological replicates.

### Phagocytosis Assay

BV-2 cells were transfected as described previously. Six hours before starting the phagocytosis assay, 12,000 cells per well on 96-well plates were plated in BV-2 cell medium. pH-sensitive red fluorescent bioparticles (P35364; Invitrogen) were added to the cells 24 or 48 h after transfection. Cells without particles were used for background subtraction in the red channel. Untransfected cells were used for background subtraction in the green channel (ZsGreen1). Microscopy images were acquired with 10 × objective from brightfield, green, and red fluorescent channels (300 ms acquisition time) every 15 min using IncuCyte S3 (Essen BioScience). Three (48 h samples) or four (24 h samples) independent experiments were conducted. Per experiment, four images per well of three technical replicates per biological replicate were acquired. For analysis, the IncuCyte software (v2019B) was used. For the green and red channels, Top-Hat segmentation (radius 100 μm, two GCU/RCU thresholds) and an area filter of 70 μm^2^ for the green channel were used. To estimate the phagocytic capacity of only transfected cells, the size of the red fluorescent area within the green fluorescent area was calculated and normalized to total green fluorescent area for each image. Absolute values were calculated for each biological replicate and pooled from three to four independent experiments.

### Cell Viability Assay

To measure cell viability 24 h after transfection, BV-2 cells were scraped, centrifuged at 800 × *g* for 3 min, resuspended in cell culture medium, and diluted in an equal volume of 0.4% trypan blue (T10282; Invitrogen). The numbers of trypan blue–positive cells and the total number of cells were counted using a hemocytometer (DHC-B01; NanoEnTek). The average number of trypan blue–positive cells was normalized to total cell number from eight technical replicates of two biological replicates per experiment. To measure cell viability 48 h after transfection, the BV-2 cell medium was replaced 2 and 24 h after transfection. Cells were maintained for an additional 24 h at +37°C and 5% CO_2_. To take both attached and floating cells (which may represent either activated or dead cells) into account, BV-2 cells were scraped in their conditioned medium, diluted in additional BV-2 medium (in total 3 mL per well), and stained and counted as described previously.

### Statistical Analyses and Data Presentation

Shapiro–Wilk test was used to test if data points were normally distributed. To test for equality of variances, Levene's test was used. To test for significance between two independent groups, two-tailed independent samples *t*-test (for normally distributed data and equal variances) or Mann–Whitney *U*-test was performed. In groups not following Gaussian distribution, significance between more than two independent groups was tested by Kruskal–Wallis test followed by Dunn's multiple comparison test. To assess statistical independence between categorical variables, Fisher's exact test was used. Kruskal–Wallis test was performed using GraphPad Prism software (version 8.2.0 for Windows; GraphPad Software, San Diego, California USA). For all other tests, SPSS version 25.0.0.1 (IBM, Armonk, NY, USA) was used. *P*-values are indicated above the compared groups. *P* < 0.05 were considered statistically significant. Graphs were drawn using the GraphPad Prism software (version 8.2.0 for Windows). The number of biological replicates per experiment and the number of independent experiments are indicated in each figure legend. The replicates were considered as biological replicates when cells of the same passage were plated and individually transfected or treated in separate wells. Cells cultured and treated within the same well were considered technical replicates. Microscopy images shown in the article were processed using Zen software (Zeiss, version 2.3, blue edition; Carl Zeiss Microscopy GmbH). Images were scaled using GIMP (version 2.10.).

## Results

### BV-2 Cells Expressing the *C9orf72* HRE Show Strong Expression of Different Dipeptide Repeat (DPR) Proteins but Not Sense RNA Foci

To assess whether microglial cells harboring the *C9orf72* HRE show RNA foci or DPR proteins, BV-2 cells were transiently transfected with plasmids encoding the *GGGGCC*-motif 66 times (66R) or 2 times (2R; control). These plasmids have been previously used to overexpress the *GGGGCC*-motif *in vitro* in U251 glial fibrillary acidic protein–positive glioma cells (45) or *in vivo* in mouse brain via intraventricular adeno-associated virus (AAV)–mediated delivery (44). In the latter study, the 66R expression led to the generation of sense RNA foci and expression of different DPR proteins (poly-GA, poly-GP, and poly-GR) in the CNS, neurodegeneration in specific brain areas, as well as behavioral deficits similar to the patients carrying the *C9orf72* HRE (44).

**Figure 1:**
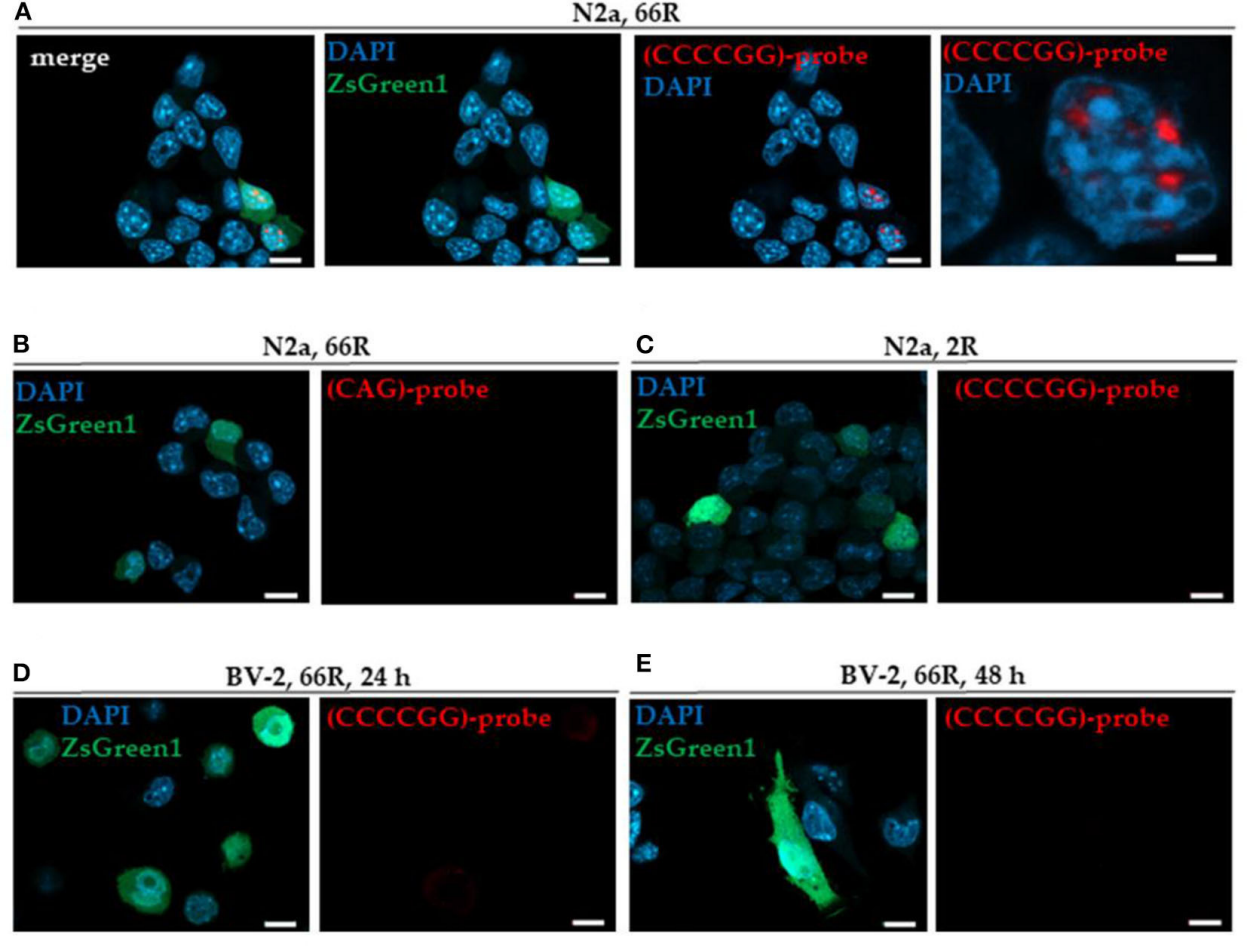
BV-2 cells harboring the *C9orf72* hexanucleotide repeat expansion do not form intranuclear sense RNA foci. Cells were transiently co-transfected with 2R or 66R in combination with a plasmid coding for ZsGreen1 (green). Nuclei were stained with DAPI (blue). Sense RNA foci were detected via FISH using TYE 563-(CCCCGG)_3_ LNA probes (red) in N2a cells transfected with 66R plasmid **(A)** but not in N2a cells transfected with 2R plasmid **(C)**. No fluorescent signal was detectable when using TYE 563-(CAG)_6_ negative control probes, demonstrating the specificity of TYE 563-(CCCCGG)_3_ probes **(B)**. Sense RNA foci were not detected in BV-2 cells transfected with 66R plasmid 24 or 48 h after transfection **(D,E)**. Representative images from three **(D)** and two **(E)** independent cell culture experiments are shown. Scale bar = 10 μm **(A–E)**; scale bar = 2 μm (**A**, right picture). DAPI, 4′,6-diamidino-2-phenylindole; FISH, fluorescence *in situ* hybridization; LNA, locked nucleic acid; ZsGreen1, *Zoanthus* sp. green fluorescent protein 1.

In this study, we used transfection of N2a neuroblastoma cells, a neuronal cell line, as a positive control to detect sense RNA foci. We found that overexpression of the 66R but not 2R in N2a cells led to the formation of sense RNA foci (Figures 1A,C), as detected by FISH and TYE 563-(CCCCGG)_3_ probe targeted against the expanded *GGGGCC* repeats. Notably, FISH with the TYE 563-(CAG)_6_ negative control probe showed no signal in 66R-transfected N2a cells, demonstrating the specificity of the TYE 563-(CCCCGG)_3_ probe against the expanded *GGGGCC* repeats (Figure 1B). In contrast to the N2a cells, however, 66R-transfected BV-2 cells did not show sense RNA foci 24 or 48 h after transfection using the TYE 563-(CCCCGG)_3_ probe (Figures 1D,E).

**Figure 2:**
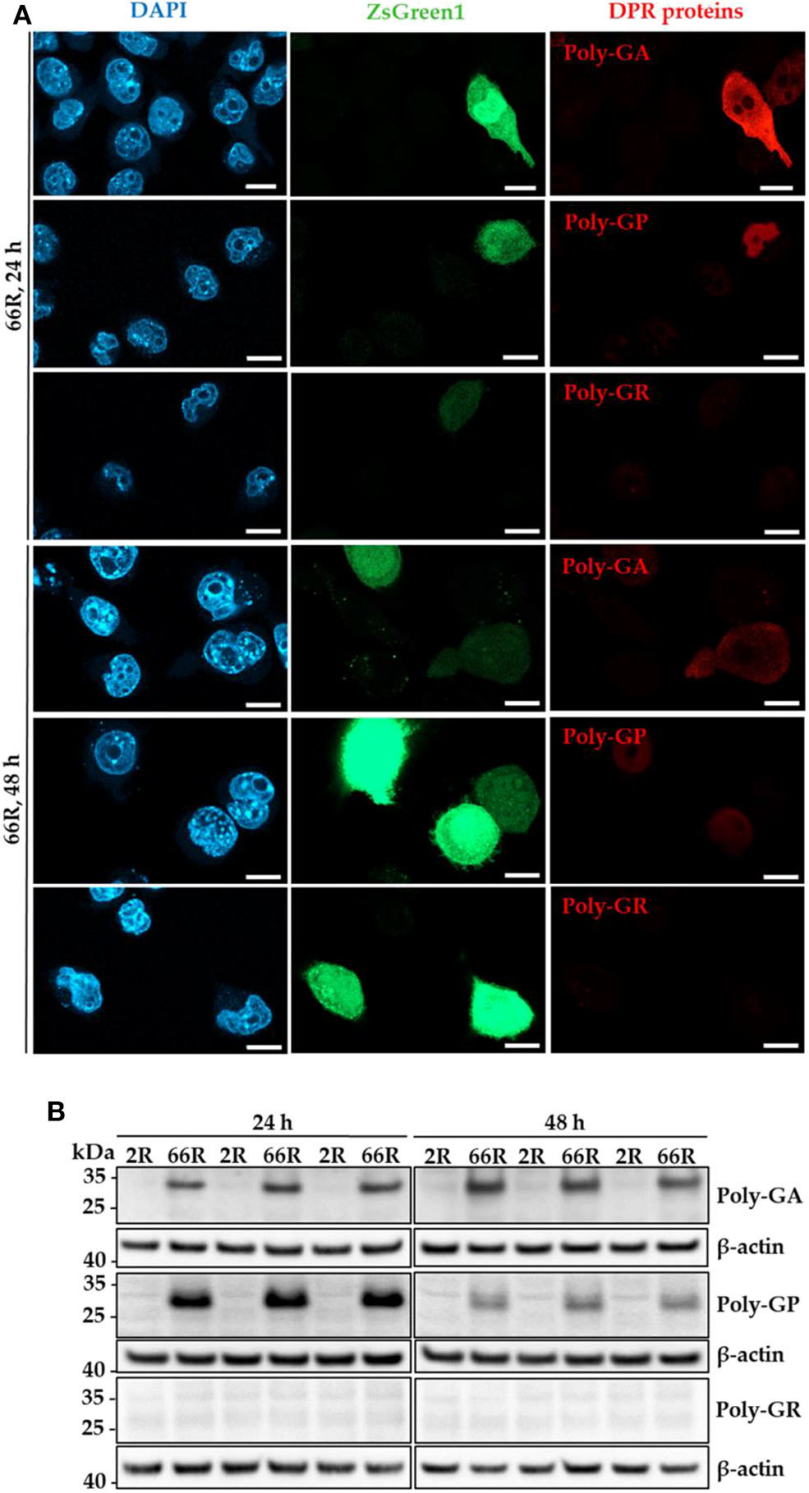
Specific DPR proteins are abundantly expressed in BV-2 cells harboring the *C9orf72* hexanucleotide repeat expansion. Poly-GA, poly-GP, and poly-GR levels were assessed 24 and 48 h after transient transfection of BV-2 cells with either 66R plasmid or control plasmid (2R) with (ICC) or without (WB) a plasmid coding for ZsGreen1 (ICC, green). **(A)** Representative microscopy images from two (24 h) or three (48 h) independent experiments of BV-2 cells overexpressing 66R plasmids and stained for poly-GA, poly-GR, or poly-GP (red). Nuclei were stained with DAPI (blue). Scale bar = 10 μm. Negative control images of 2R-transfected BV-2 cells and primary antibody control stainings are presented in Supplementary Figures 2, 3. **(B)** Representative Western Blot images of four independent experiments are shown, indicating high levels of poly-GA and poly-GP, but not poly-GR in BV-2 cells. DAPI, 4′,6-diamidino-2-phenylindole; DPR, dipeptide repeat; ICC, immunocytochemistry; kDa, kilodalton; WB, Western blotting; ZsGreen1, *Zoanthus* sp. green fluorescent protein 1.

Next, we investigated whether the 66R-overexpressing BV-2 cells display DPR proteins. Immunocytochemistry (ICC) and Western blotting (WB) showed that poly-GA and poly-GP proteins were present in BV-2 cells 24 and 48 h after transfection with the 66R plasmids (Figures 2A,B). No signal was detectable in BV-2 cells transfected with the 2R control plasmid or in 66R-expressing BV-2 cells incubated without the primary antibodies, indicating the specificity of the signal (Supplementary Figures 2, 3). Whereas, the poly-GA immunoreactivity was distributed throughout the whole cell body (cytoplasm and nuclei), the poly-GP signal was restricted to nuclei. No aggregates or inclusions of DPR proteins were detected (Figure 2A). In the WB analysis, the DPR protein-specific bands were detected at abundant levels at the molecular weights between 25 and 35 kDa in 66R-expressing BV-2 cells (Figure 2B). No high molecular weight bands specific to 66R-expressing cells were observed (Supplementary Figure 4). No poly-GR immunoreactivity was detected using WB or ICC (Figures 1A,B). Similar to the BV-2 cells, we have also observed poly-GA and poly-GP, but not poly-GR, DPR proteins in N2a cells transfected with 66R by WB and ICC (unpublished data), confirming that these are the main DPR protein species produced at detectable levels from the 66R construct. These results altogether show that BV-2 cells harboring the *C9orf72* repeat expansion produce soluble poly-GA and poly-GP but not poly-GR DPR proteins or sense RNA foci.

### BV-2 Cells Expressing the *C9orf72* HRE Show a Transient Cytoplasmic Translocation and Increased Levels of TDP-43

**Figure 3:**
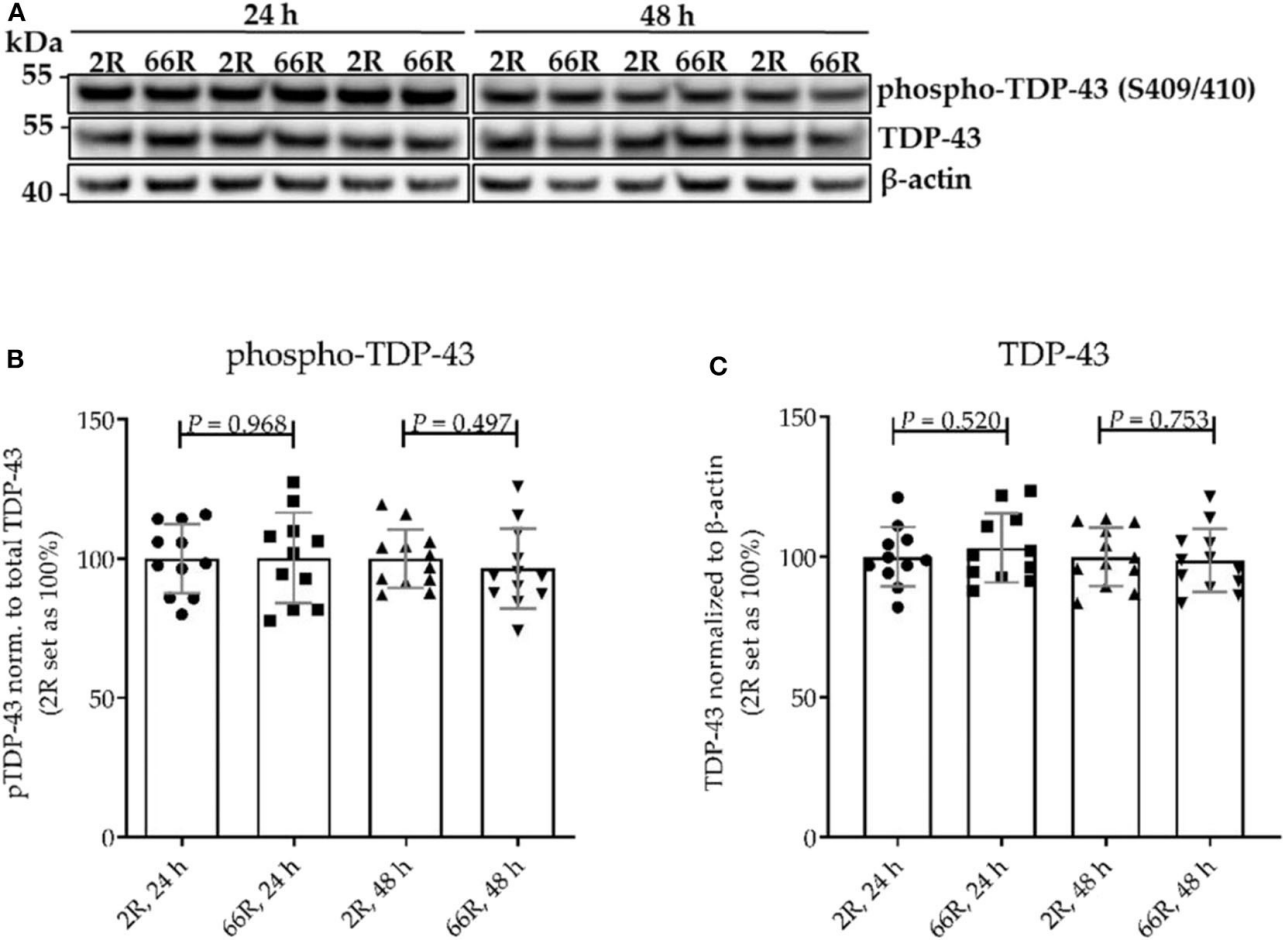
The levels of TDP-43 and phosphorylated TDP-43 are not altered in BV-2 cells harboring the *C9orf72* hexanucleotide repeat expansion. TDP-43 phosphorylation (p-TDP-43) at site S409/S410 was assessed 24 and 48 h after transfection of BV-2 cells with either 66R plasmid or control plasmid (2R). **(A)** Representative Western blot images of four independent experiments are shown. Quantification shows mean ± SD of p-TDP-43 levels normalized to total TDP-43 levels **(B)** and total TDP-43 levels normalized to those of β-actin **(C)** from four independent experiments each with two to three biological replicates. For each experiment and time point, 2R was set as 100%. Two-tailed independent samples *t*-test **(B,C)** was performed. *n* = 11, *df* = 20 (24 h); *n* = 12, *df* = 22 (48 h). *df*, degrees of freedom; kDa, kilodalton; TDP-43, TAR DNA-binding protein 43.

**Figure 4:**
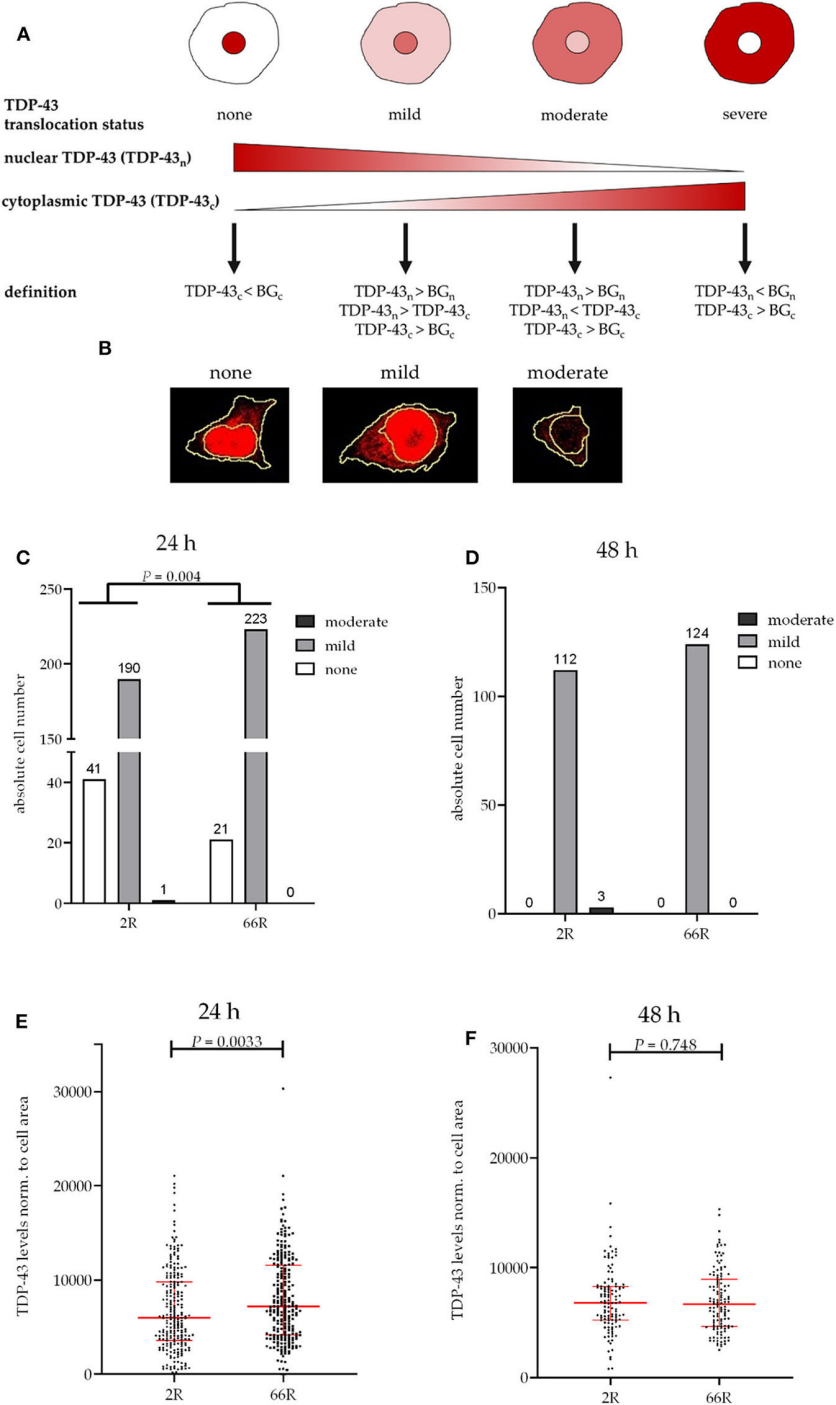
TDP-43 subcellular localization is transiently altered in BV-2 cells harboring the *C9orf72* hexanucleotide repeat expansion. TDP-43 translocation from the nucleus (_n_) to the cytoplasm (_c_) was quantified based on Alexa Fluor 647 intensity (secondary antibody against TDP-43) using fluorescence microscopy and categorized into different stages **(A,B)** in BV-2 cells harvested 24 h **(C,E)** or 48 h **(D,F)** after transfection with either 2R or 66R plasmids in combination with a plasmid coding for ZsGreen1. Nuclear and cytoplasmic background (BG) intensity values were obtained from ZsGreen1-positive 2R or 66R cells immunostained without primary antibody. TDP-43 translocation is shown as absolute cell counts **(C,D)**; total cell number analyzed for 24 h/48 h 2R = 232/115, 66R = 244/124. SUM intensity of Alexa Fluor 647 in the whole cell was normalized to whole cell area **(E,F)**. Data are shown as median ± interquartile range **(E,F)**. Data are shown from two (48 h) or three (24 h) independent cell culture experiments. Fisher's exact test **(C)** and Mann–Whitney *U*-test **(E,F)** were used to test for statistical significance. BG, background; DAPI, 4′,6-diamidino-2-phenylindole; TDP-43, TAR DNA-binding protein 43; ZsGreen1, *Zoanthus* sp. green fluorescent protein.

TDP-43 is an RNA-binding protein, which shuttles between nucleus and cytosol (46). Hyperphosphorylation of TDP-43 and accumulation of cytoplasmic TDP-43 have been observed in the CNS of patients with FTLD and ALS, including *C9orf72* HRE carriers (40, 47–49). These features have been described in glial cells of *C9orf72* HRE carriers to some extent as well, but without further differentiation of the subtype, i.e., astrocytes, oligodendrocytes, or microglia (40, 48). Phosphorylation, total levels, and subcellular distribution of TDP-43 were examined in BV-2 cells 24 and 48 h after transfection with 2R or 66R plasmid to assess whether the expression of the *C9orf72* HRE leads to TDP-43 pathology in microglial cells (Figures 3, 4). To assess TDP-43 localization in the cytoplasm and nucleus of 2R- and 66R-transfected BV-2 cells, co-transfection of 2R or 66R with a ZsGreen1-encoding plasmid was conducted. According to ICC, 24 h after transfection 94% and 48 h after transfection 88% of cells expressing ZsGreen1 were also positive for poly-GA. No cross-reactivity between poly-GA and TDP-43 secondary antibodies was observed (Supplementary Figure 5). To assess the degree of TDP-43 translocation from nucleus to cytoplasm, four different TDP-43 distribution types were categorized depending on the ratio of nuclear to cytoplasmic TDP-43 levels (Figures 4A,B).

No difference in TDP-43 phosphorylation or total TDP-43 levels between 2R- and 66R-expressing BV-2 cells could be detected in either time point (Figure 3; Supplementary Figure 6). Twenty-four hours after transfection, BV-2 cells expressing the 66R plasmid displayed a significantly decreased number of cells with TDP-43 localized in the nucleus only (no cytoplasmic translocation) and an increased number of cells with mild TDP-43 cytoplasmic translocation as compared with 2R cells (Figure 4C). Forty-eight hours after transfection, both 2R- and 66R-expressing cells showed a similar mild TDP-43 cytoplasmic translocation, and there were no differences between the cells in TDP-43 subcellular localization (Figure 4D). In addition, the total TDP-43 levels in ZsGreen1-positive cells were quantified. Twenty-four hours after transfection, 66R-expressing cells showed higher TDP-43 levels within the whole cell body as compared with 2R-expressing cells (Figure 4E). Forty-eight hours after transfection, no difference in total TDP-43 levels between 2R- and 66R-expressing cells could be detected (Figure 4F). Together, these data suggest that a transient cytoplasmic translocation and upregulation of TDP-43 levels takes place in BV-2 cells after introducing the *C9orf72* HRE.

### Expression of the *C9orf72* HRE Reduces Levels of Polyubiquitinylated Proteins

**Figure 5:**
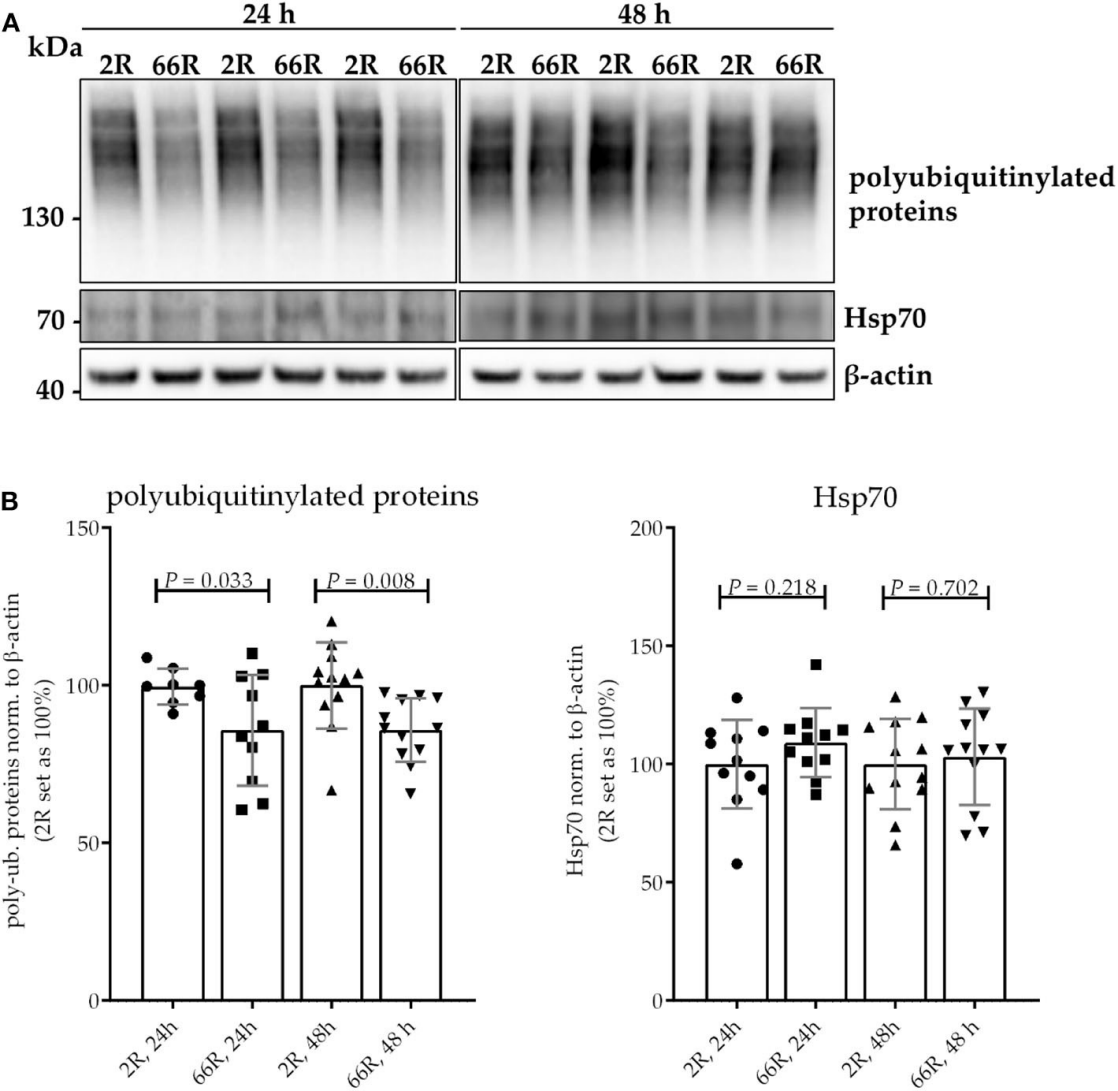
BV-2 cells expressing the *C9orf72* hexanucleotide repeat expansion show lower levels of polyubiquitinylated proteins but equal levels of Hsp70. Representative blot images **(A)** and corresponding quantifications **(B)** from four independent cell culture experiments. Protein samples were harvested from BV-2 cells expressing the 2R or 66R plasmid for 24 and 48 h. Polyubiquitinated proteins and Hsp70 levels were normalized to those of β-actin. 2R was set as 100% for each time point and experiment. Data are shown as mean ± SD. Two-tailed independent samples *t*-test was used; *n* = 11, *df* = 20 (24 h) (for polyubiquitinated proteins, 24 h: *n* = 9/10 (2R/66R), no equal variances were assumed, *df* = 10,923); *n* = 12, *df* = 22 (48 h). df, degrees of freedom; kDa, kilodalton; Hsp70, heat shock protein 70.

Protein homeostasis is ensured at the cellular level through the function of the ubiquitin–proteasome system (UPS) and chaperone proteins, such as heat shock proteins (Hsps). Hsps facilitate the correct folding of proteins and can direct misfolded proteins to degradation through the UPS or autophagy. Inefficient clearance of misfolded proteins is associated with neurodegeneration (50). To assess whether expression of the *C9orf72* HRE leads to changes in the UPS or Hsps in microglial cells, levels of polyubiquitinylated proteins Hsp25 and Hsp70 were examined using WB. Twenty-four and 48 h after transfection, 66R-expressing BV-2 cells showed lower levels of polyubiquitinylated proteins as compared with 2R-expressing cells (Figures 5A,B; Supplementary Figures 7A,B). Hsp25 was not present in the BV-2 cells at detectable levels (data not shown). No difference in levels of Hsp70 could be detected between 2R- and 66R-expressing cells neither 24 nor 48 h after transfection (Figures 5A,B; Supplementary Figures 7C,D). These results suggest that expression of the *C9orf72* HRE might affect the UPS function without altering Hsp levels.

### Expression of the *C9orf72* HRE Does Not Influence Viability of BV-2 Cells

**Figure 6:**
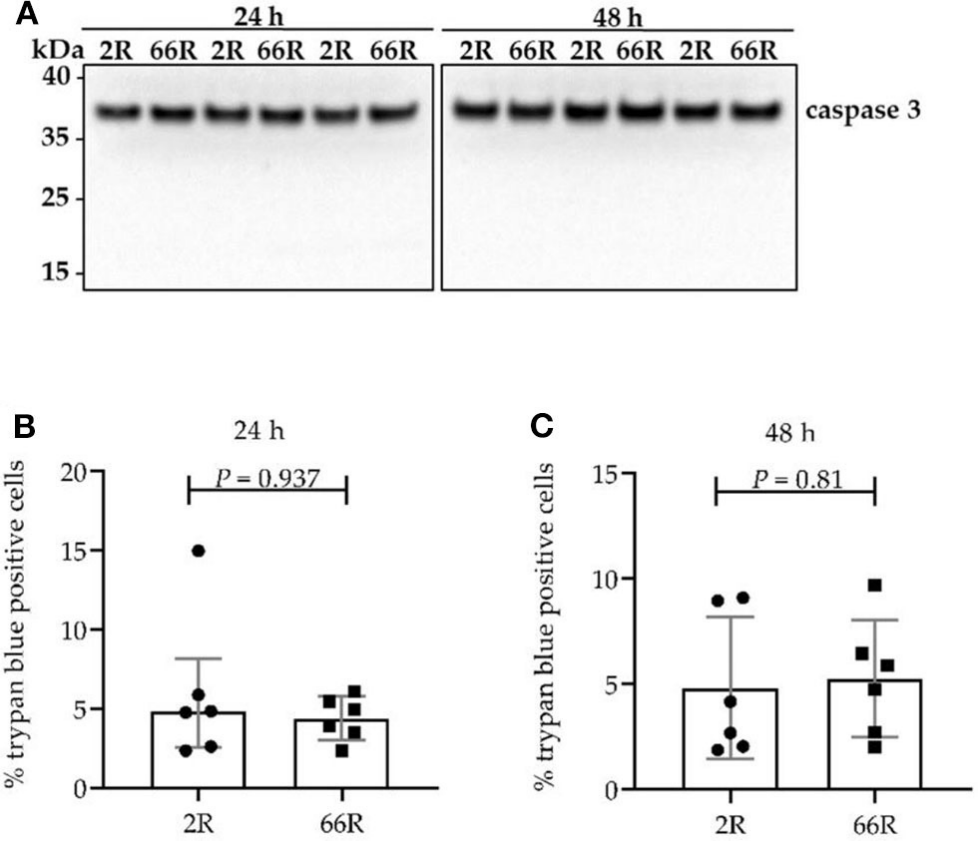
Cell viability is not affected in BV-2 cells harboring the *C9orf72* hexanucleotide repeat expansion. Caspase 3 cleavage **(A)** was assessed 24 and 48 h after transfection with 66R or control (2R) plasmids by Western blotting. Cleaved caspase 3 fragments would appear at 17 and 19 kDa. Representative blot images from four independent experiments are shown. Plasma membrane integrity was measured 24 h **(B)** and 48 h **(C)** after transfection of BV-2 cells with 66R or control (2R) plasmids by counting number of trypan blue–positive cells and normalization to the total cell number. Data are presented as median ± interquartile range **(B)** and mean ± SD **(C)** of the percentage of trypan blue–positive cells normalized to total cell number from three independent experiments each with two biological replicates. Mann–Whitney *U*-test **(B)** and two-tailed independent samples *t*-test **(C)** were performed; *n* = 6, *df* = 10. df, degrees of freedom; kDa, kilodalton.

Caspase 3 cleavage from the full-length protein (proenzyme) into the active form and loss of plasma membrane integrity are considered common signs of cell death (51). To elucidate whether BV-2 cells expressing 66R undergo caspase 3–dependent apoptosis or necrosis, caspase 3 cleavage was assessed using WB and plasma membrane integrity loss using trypan blue staining. Twenty-four and 48 h after transfection, BV-2 cells showed no caspase 3 cleavage products (Figure 6A). Moreover, no difference in plasma membrane integrity (Figures 6B,C) could be detected when comparing 2R- with 66R-expressing BV-2 cells. These findings indicate that BV-2 cells harboring the *C9orf72* HRE do not undergo apoptosis or necrosis even though they abundantly express DPR proteins.

### Expression of the *C9orf72* HRE Slightly Alters p62 and LAMP2A Levels but Not Phagocytosis in BV-2 Cells

Whereas, autophagy is the process through which intracellular components (ranging from proteins to cell organelles) are degraded, phagocytosis refers to the ingestion and digestion of extracellular cell debris and pathogens. Both pathways share a common set of signaling molecules (such as reactive oxygen species, redox equivalents) and proteins. These proteins include the lysosomal lysosome-associated membrane proteins (LAMP)-1 and−2, which fuse with vesicles formed during phagocytosis/autophagy to provide an acidic environment for proteolytic degradation. Whereas, this step occurs later in the cascade, two other key proteins are involved in the induction of autophagy. During the formation of a double-membrane vesicle, also called the phagophore, microtubule-associated protein 1 light chain 3 beta (LC3B) I is lipidated and incorporated into the phagophore membrane. The lipidated form of LC3BI is termed LC3BII, and the conversion of LC3BI to LC3BII marks the initiation of autophagy. The cargo to be degraded is targeted to the phagophore via adaptor molecules (52). Degradation of one of these adaptor proteins, p62, is used as another marker for autophagy. Also, it has been proposed that the C9orf72 proteins are involved in the initial phase of autophagy (53, 54).

**Figure 7:**
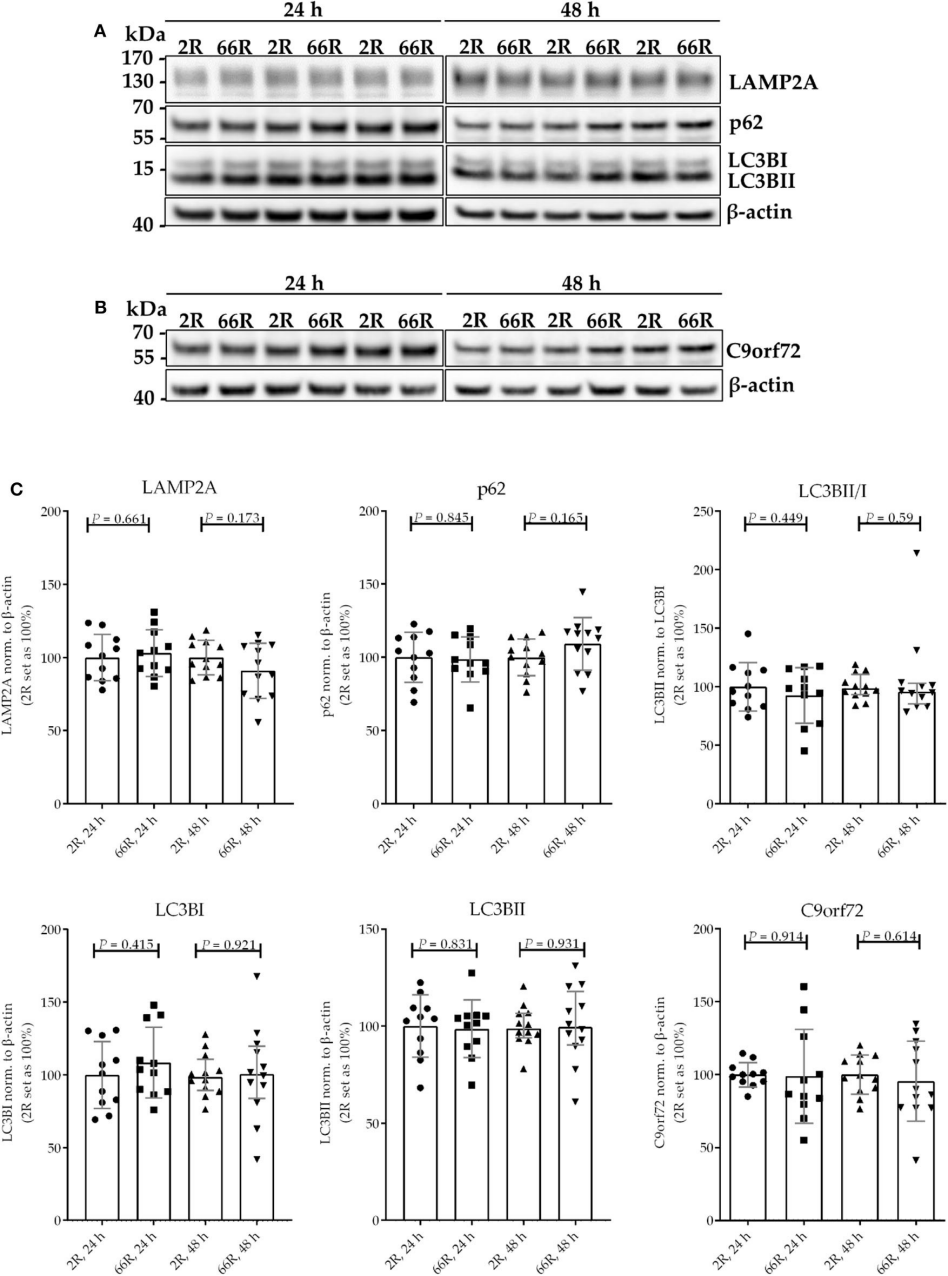
BV-2 cells expressing the *C9orf72* hexanucleotide repeat expansion do not show altered basal autophagy. Representative blot images **(A,B)** and corresponding quantifications **(C)** from four independent cell culture experiments. Protein samples were harvested from BV-2 cells expressing the 2R or 66R plasmid for 24 and 48 h. P62, LAMP2A, LC3BI, LC3BII, and C9orf72 levels were normalized to those of β-actin and additionally LC3BII levels to those of LC3BI. 2R was set as 100% for each time point and experiment. Data are shown as median ± interquartile range (LC3BII/LC3BI, 48 h) or as mean ± SD. To compare LC3B conversion between 2R and 66R 48 h after transfection, Mann–Whitney *U*-test was used. Otherwise, two-tailed independent samples *t*-test was used; *n* = 11, *df* = 20 (24 h); *n* = 12, *df* = 22 (48 h)—for C9orf72 data, no equal variances were assumed, *df* = 11.355 (24 h), *df* = 16.021 (48 h). df, degrees of freedom; kDa, kilodalton; LAMP2A, lysosome-associated membrane protein 2 a; LC3B, microtubule-associated protein 1 light chain 3 beta.

**Figure 8:**
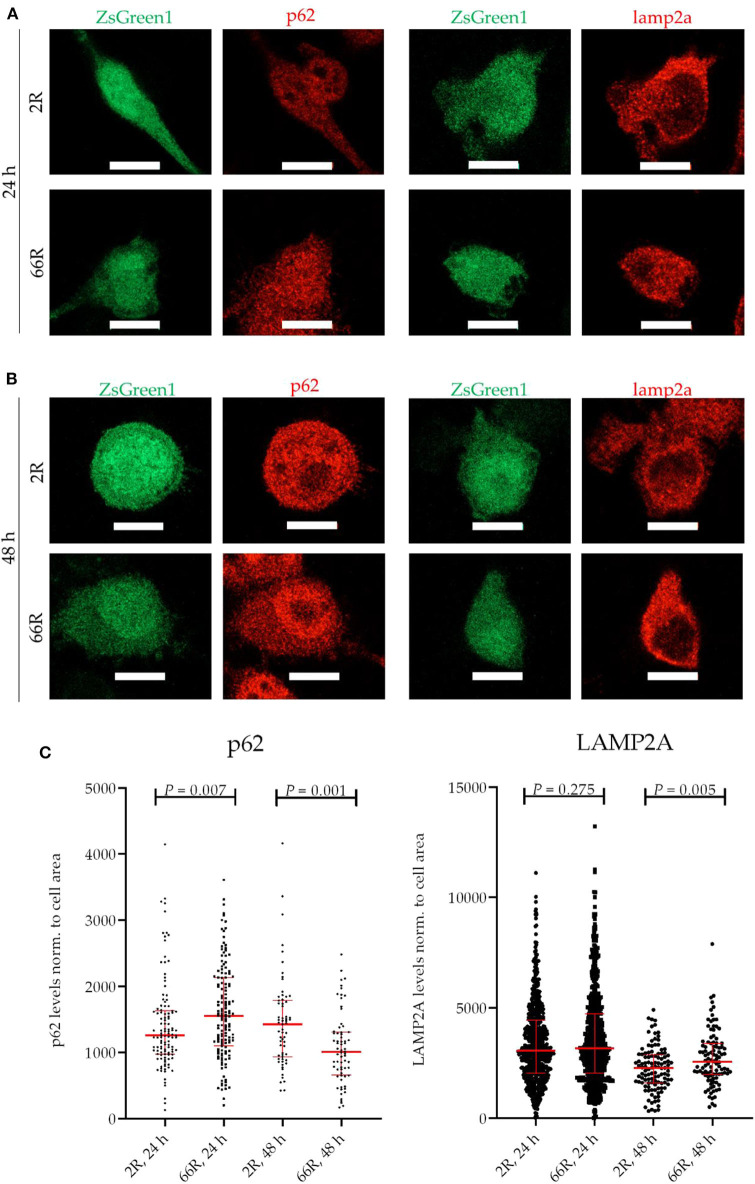
BV-2 cells expressing the *C9orf72* hexanucleotide repeat expansion show altered levels of p62 and LAMP2A. Representative microscopy images **(A,B)** and corresponding quantifications **(C)** from two (p62) and three (LAMP2A) independent cell culture experiments. P62 and LAMP2A (red) levels were quantified on the basis of Alexa Fluor 568 intensity (secondary antibody) using fluorescence microscopy. Data were normalized to cell area. BV-2 cells were harvested 24 h **(A,C)** or 48 h **(B,C)** after transfection with either 2R or 66R plasmids in combination with a plasmid coding for ZsGreen1. Only ZsGreen1 (green) positive cells were analyzed. Scale bar = 10 μm. Data are shown as median ± interquartile range. Mann–Whitney *U*-test was performed; for p62: *n* = 115/144 (2R/66R; 24 h), *n* = 63/68 (2R/66R; 48 h); for LAMP2A: *n* = 636/585 (2R/66R; 24 h), *n* = 113/102 (2R/66R; 48 h). LAMP2A, lysosome-associated membrane protein 2 a; ZsGreen1, *Zoanthus* sp. green fluorescent protein.

Because *C9orf72* HRE carriers show accumulation of p62 in the brain, which might indicate compromised autophagy (40), we investigated whether the levels of proteins involved in autophagy/phagocytosis are altered upon *C9orf72* HRE expression. To assess autophagy in BV-2 cells, p62 levels (normalized to β-actin), LC3BI and LC3BII levels (normalized to β-actin), and LC3BI to LC3BII conversion were measured via WB. Twenty-four and 48 h after transfection, no differences in p62, LC3BI, or LC3BII levels or LC3B conversion could be detected between the 66R- and 2R-expressing BV-2 cells (Figure 7; Supplementary Figure 8). Also, no alterations in C9orf72 (55 kDa) and LAMP-2A levels were detected 24 or 48 h after transfection by WB (Figure 7; Supplementary Figure 8). To quantify p62 and LAMP2A levels at a single-cell level, co-transfection of 2R or 66R with a ZsGreen1-encoding plasmid was conducted, and p62 and LAMP2A levels were measured in ZsGreen1-positive cells using ICC and confocal microscopy. Whereas, the 66R-transfected cells showed mildly increased levels of p62 at 24 h after transfection, the levels were lower at 48 h after transfection as compared with 2R cells (Figure 8). No difference in the LAMP2A levels between the 2R- and 66R-transfected cells could be detected 24 h after transfection. However, 48 h after transfection, 66R-transfected cells showed slightly higher levels of LAMP2A compared with 2R-transfected cells (Figure 8).

**Figure 9:**
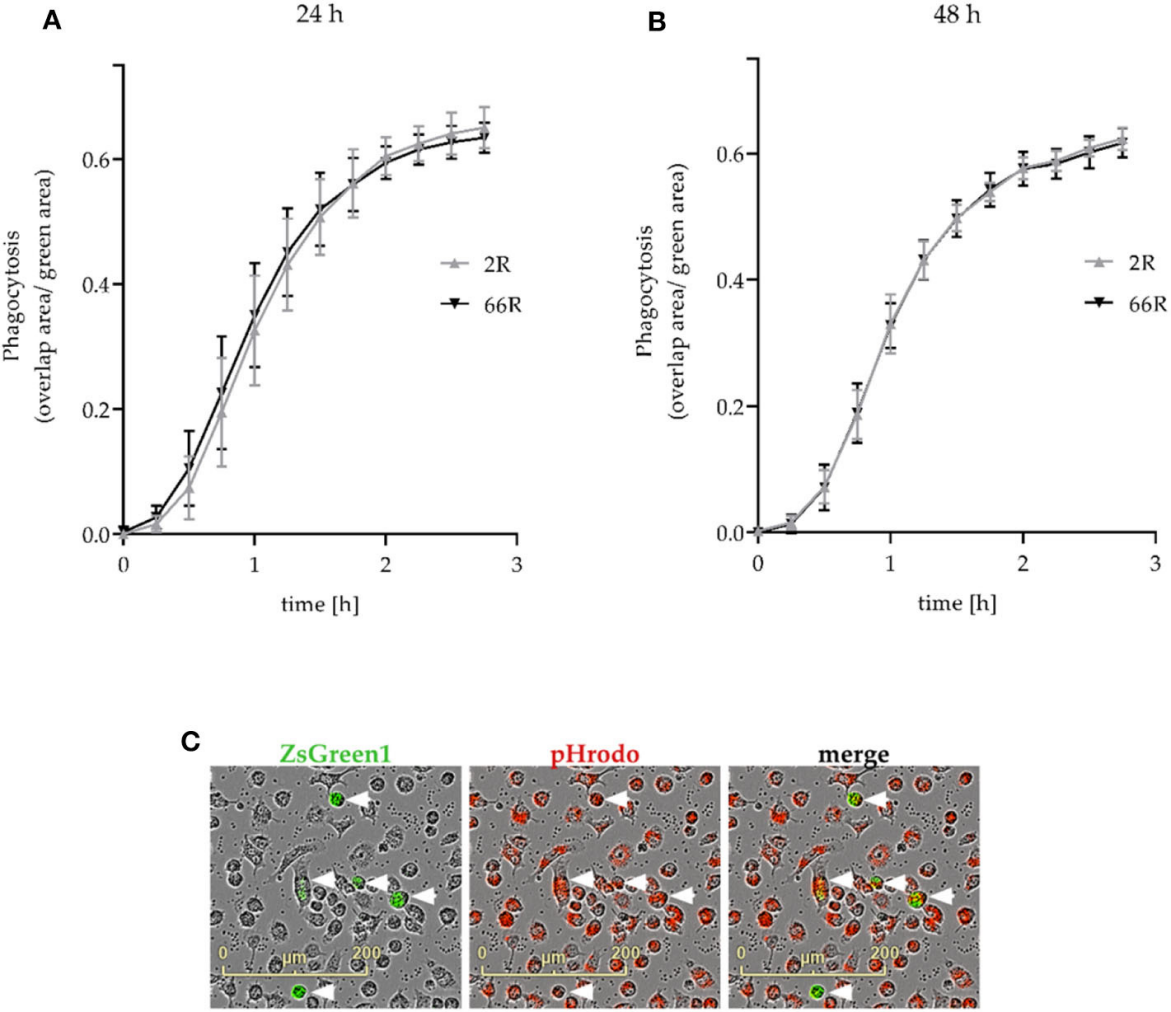
Phagocytosis is not affected in BV-2 cells expressing the *C9orf72* hexanucleotide repeat expansion. Phagocytosis was measured 24 h **(A)** and 48 h **(B)** after the transfection of BV-2 cells with 2R or 66R in combination with a ZsGreen1-encoding plasmid. BV-2 cells were incubated with pHrodo Red Zymosan Bioparticle Conjugates (red), and phagocytosis was monitored over a 3-h timescale. **(C)** Representative microscopy images of 66R-transfected BV-2 cells expressing ZsGreen 1 (green) and ingesting pHrodo particles (red) are indicated by arrowheads. The area of red fluorescence within the green fluorescence area (overlap area) was normalized to the total size of the green fluorescent area. The time course is shown as mean ratio ± SD. Three **(B)** and four **(A)** independent cell culture experiments each with three biological replicates were performed. To test for statistical differences between 2R- and 66R-transfected cells for each time point, two-tailed independent samples *t*-test (for normally distributed data) or Mann–Whitney *U*-test was used, *n* = 12 (per treatment, 24 h), *df* = 22; *n* = 9 (48 h, time points 0–2 h), *df* = 16; *n* = 6 (48 h, time points 2.25–2.75 h), *df* = 10. No significant differences (*P*-value below 0.05) could be detected between 2R and 66R samples at any time point. df, degrees of freedom; ZsGreen1, *Zoanthus* sp. green fluorescent protein.

Next, we assessed whether phagocytosis, one of the key functions of microglial cells, is affected upon expression of the *C9orf72* HRE. We used co-transfection of 2R or 66R and ZsGreen1 plasmids and live cell imaging to measure phagocytosis in transfected cells (see section Phagocytosis Assay; Figure 9C). Twenty-four and forty eight hour after transfection, no difference in the phagocytic activity of BV-2 cells expressing 66R could be detected as compared with 2R-expressing cells at any time point measured (Figures 9A,B). These results show that expression of the *C9orf72* HRE in BV-2 microglial cells does not change their phagocytic capability.

### Response to LPS/IFNγ Treatment Is Not Affected by the Expression of the *C9orf72* HRE in BV-2 Cells

**Figure 10:**
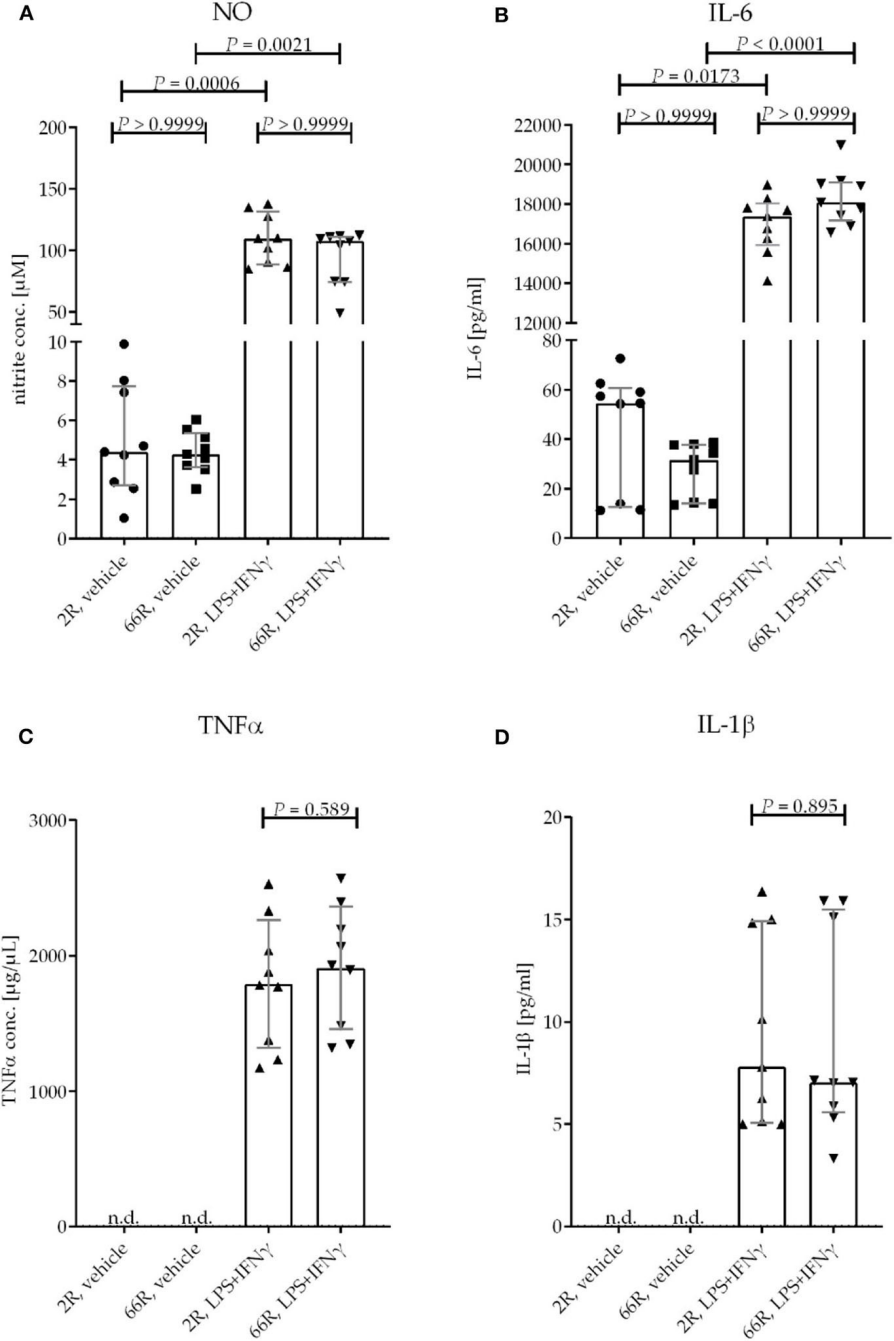
BV-2 cells expressing the *C9orf72* hexanucleotide repeat expansion do not show altered response to an inflammatory stimulus by LPS and IFNγ. BV-2 cells were transfected with 2R or 66R plasmids and treated 24 h after transfection with 200 ng/mL LPS and 20 ng/mL IFNγ or vehicle (DPBS) for 24 h. Nitric oxide (NO) levels from the media were measured using Griess reaction. TNFα, IL-1β, and IL-6 concentrations from medium samples were measured by ELISA. Data points are shown as absolute values without normalization from three independent experiments, including three biological replicates per experiment. Data are shown as median ± interquartile range **(A,B,D)** and multiplicity-adjusted *P*-values **(A,B)** or as mean ± SD **(C)**. To test for statistical significance, Kruskal–Wallis test followed by Dunn's multiple comparison test **(A,B)**, two-tailed independent samples *t*-test **(C)**, or Mann–Whitney *U*-test **(D)** were performed. For each time point and treatment: *n* = 9, *df* = 32 **(A,B)**; *n* = 9, *df* = 16 **(C,D)**. df, degrees of freedom; DPBS, Dulbecco's phosphate-buffered saline; IFN, interferon; IL-1β, interleukin 1β; IL-6, interleukin 6; LPS, lipopolysaccharide; n.d., not detectable; NO, nitric oxide; TNF, tumor necrosis factor.

One of the key functions of microglia is the ability to react to environmental stimuli. *In vitro* experiments have shown that lipopolysaccharide (LPS) and interferon (IFN) γ cause increased production and secretion of tumor necrosis factor alpha (TNFα), interleukin (IL) 1β and 6, and nitric oxide (NO) by microglia (55–57). To assess whether the expression of the *C9orf72* HRE influences the response of microglia to LPS and IFNγ, BV-2 cells were treated concomitantly with both at 24 h after transfection for 24 h. Extracellular levels of TNFα, IL-6, IL-1β, and NO (measured indirectly as nitrite) were elevated in both 2R- and 66R-expressing BV-2 cell media upon treatment. However, no difference in the levels of NO, TNFα, IL-1β, or IL-6 after the LPS/IFNγ treatment could be detected between 2R- and 66R-transfected BV-2 cells. In addition, no differences in NO and IL-6 levels in the media of 66R cells compared with and 2R cells could be detected in vehicle-treated cells (Figures 10A–D). These results indicate that expression of the *C9orf72* HRE does not lead to activation of BV-2 cells nor does it impair their ability to respond to LPS and IFNγ treatment and subsequent production of inflammatory modulators.

### Expression of the *C9orf72* HRE Does Not Trigger Disease-Associated Microglia RNA Signature in BV-2 Cells

Previous studies have described a subgroup of microglia termed disease-associated microglia (DAM) based on their characteristic RNA profile in the brain of, e.g., Alzheimer's disease model mice and patients. DAM derive from homeostatic microglia through two stages. The first stage is considered independent of triggering receptor expressed on myeloid cells 2 (Trem2), whereas the second stage is Trem2 dependent. Each stage is characterized by a specific mRNA profile, where homeostatic microglial genes are downregulated, but genes involved in phagocytosis, lipid metabolism, and lysosomal pathways are upregulated. Whereas, some subgroups of DAM are considered functionally compromised and eventually neurotoxic, others might be even neuroprotective (58–62).

**Figure 11:**
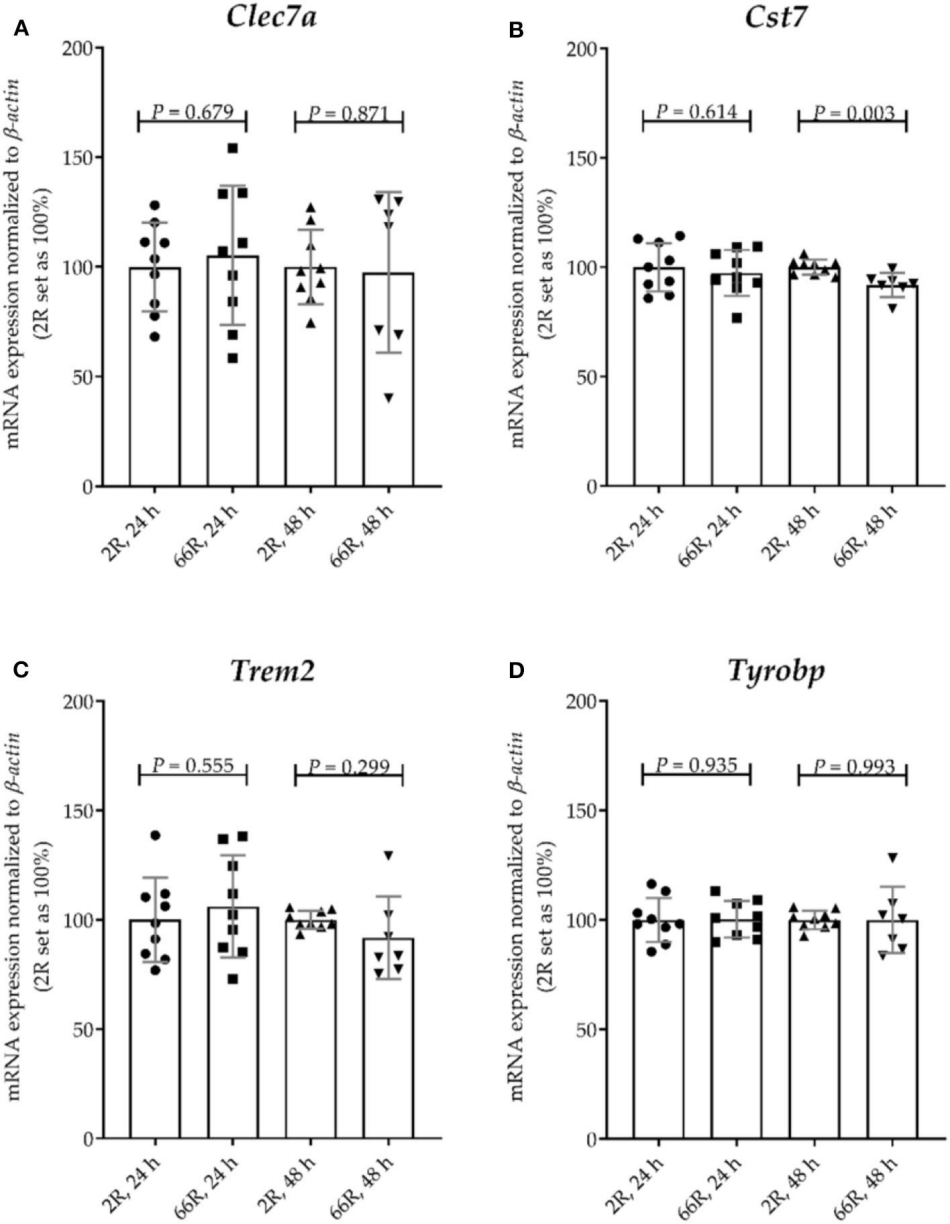
BV-2 cells expressing the *C9orf72* hexanucleotide repeat expansion do not show major changes in the expression of disease-associated microglia marker genes. BV-2 cells were transfected with 2R or 66R plasmids, and RNA samples were collected 24 or 48 h after transfection. **(A–D)** Data are shown as mean values ± SD from three independent cell culture experiments. mRNA levels were normalized to those of β*-actin*. 2R was set as 100% for each time point and experiment. To test for statistical significance, two-tailed independent samples *t*-test was performed, no equal variances were assumed for 48 h **(A,C,D)**; *n* = 7 (66R, 48 h) and otherwise *n* = 9; df (24 h) = 16, df (48 h, **A**) = 7.988, df (48 h, **B**) = 14, df (48 h, **C**) = 6.453, df (48 h, **D**) = 6.732. *Clec7a, C-type lectin domain family 7, member a*; *Cst7, cystatin F*; df, degrees of freedom; *Trem2, triggering receptor expressed on myeloid cells 2*; *Tyrobp, TYRO protein tyrosine kinase binding protein*.

To assess whether expression of the *C9orf72* HRE shifts BV-2 cells toward the DAM phenotype, mRNA expression levels of four DAM markers were examined. Whereas, *TYRO protein tyrosine kinase binding protein* (*Tyrobp*) expression is known to be elevated during stage 1, expression of *Trem2, C-type lectin domain family 7, member a* (*Clec7a*), and *cystatin F* (*leukocystatin*; *Cst7*) is increased during stage 2 (59). We found that 24 h after transfection, no changes in the mRNA profiles of these markers between 2R- and 66R-expressing BV-2 cells could be detected (Figures 9A–D). However, 48 h after transfection, 66R-expressing BV-2 cells showed slightly decreased expression of *Cst7* (Figure 11B), but no changes in the expression of *Trem2, Tyrobp*, and *Clec7a* compared with 2R-expressing cells could be detected (Figures 11A,C,D). These data suggest that the expression of the *C9orf72* HRE does not switch BV-2 cells to the DAM phenotype.

## Discussion

To date, most of the mechanistic studies related to the *C9orf72* HRE have concentrated on neurons, but there is no information on how the *C9orf72* HRE influences the phenotype or function of microglia. In this study, we aimed to address these knowledge gaps by investigating the effects of the *C9orf72* HRE on mouse BV-2 microglial cells. We tested whether the microglial cells expressing 66 *GGGGCC* repeats (66R) (44) show *C9orf72* HRE-derived pathological hallmarks and cellular downstream effects similarly to as previously reported in neurons, and whether the HRE affects microglial-specific functions.

In human *post-mortem* brains and brains of the 66R-expressing mice, sense and/or antisense RNA foci and DPR protein inclusions have been detected in neurons (11, 13, 16, 21, 33, 44). Here, we observed that BV-2 cells expressing the *C9orf72* HRE did not present detectable sense RNA foci, even though the expression of the HRE in neuronal N2a cells led to abundant formation of sense RNA foci. The RNA foci have been shown to sequester different RBPs in neurons, and this has been suggested to lead to deleterious effects as the sequestered RBPs can no longer mediate their normal functions (18, 23). The underlying reason for the absence of sense RNA foci in BV-2 cells harboring the *C9orf72* HRE is not clear but might be related to a potentially differential expression of RBPs in neuronal and microglial cells. Alternatively, microglial cells may be more effective in clearing the aggregated RNAs containing the expanded repeats or sequestered RBPs from the RNA foci compared with neuronal cells. Future investigations will confirm whether, e.g., human microglia show a similar phenotype or whether the lack of sense RNA foci is a specific feature of BV-2 cells expressing the *C9orf72* HRE.

Despite the lack of sense RNA foci, poly-GA and poly-GP DPR proteins were detectable at high levels already 24 h after introducing the *C9orf72* HRE to BV-2 cells. Neither poly-GA nor poly-GP formed cytoplasmic or nuclear inclusions or high molecular weight species, suggesting that they are present in soluble forms in BV-2 cells. Notably, the same 66R construct as used in the present study to deliver the *C9orf72* HRE led to intracellular poly-GA and poly-GP inclusions as well as soluble poly-GP when expressed in mouse neurons *in vivo* (44). In secondary cell lines, such as human embryonic kidney (HEK)293T cells and U251 cells, the here used 66R plasmid led to DPR protein-specific signals at or below 20 kDa (45). Because the expression of individual DPR proteins in HEK293T cells results in poly-GA and poly-GP inclusions (63), secondary cell line–specific metabolic differences or disparate length of the expanded repeat expressed might underlie the absence of inclusions in our model.

Cytoplasmic poly-GA proteins have been shown to impair the translocation of TDP-43 from the cytoplasm into the nucleus (64). Hence, *C9orf72* HRE-associated TDP-43 pathology might at least partially result from the presence of DPR proteins. TDP-43 pathology has been reported *in vivo* in neurons expressing 66R (44). Here, we detected a transient increase in the ratio of cytoplasmic to nuclear TDP-43 in BV-2 cells expressing *C9orf72* HRE. This transient increase coincided with increased total TDP-43 levels on the single cell level. However, there were no differences in TDP-43 phosphorylation in BV-2 cells harboring the *C9orf72* HRE compared with the control cells. Furthermore, we did not detect intracellular inclusions or high molecular weight forms of aggregated TDP-43 in BV-2 cells upon the expression of *C9orf72* HRE. The lack of a strong TDP-43 translocation phenotype in this study might be due to the short expression time of the HRE. It has been shown in some *C9orf72* HRE carriers and animal models that DPR protein pathology might also occur without TDP-43 pathology. This might either be due to the fact that DPR protein pathology precedes the TDP-43 pathology or that another independent trigger, such as cellular stress, is required for the TDP-43 pathology to develop (65–67). Indeed, some previous studies have suggested that the formation of phosphorylated TDP-43 inclusions is preceded by the presence of RNA foci and DPR protein inclusions (67–69). Thus, the lack of phosphorylated TDP-43 inclusions in our study might be due to the fact that the BV-2 cells did not harbor RNA foci or DPR protein aggregates. Experiments in other model systems, including patient-derived microglia, might shed further light on the relationship between the RNA foci, DPR proteins, and TDP-43 pathology.

Induced pluripotent stem cell–derived motor and cortical neurons of *C9orf72* HRE carriers show increased caspase 3 activation and loss of plasma membrane integrity (70). However, fibroblasts from *C9orf72* HRE carriers did not show caspase 3 activation or plasma membrane integrity loss when cultured *in vitro* (71). On the other hand, expression of poly-GA proteins (50 × poly-GA) by transient transfection in HEK293T cells or virus-mediated transduction in primary mouse neurons caused caspase 3 activation as well as plasma membrane integrity loss (33). In our model, BV-2 cells did not show signs of cell death 24 or 48 h after transfection with the *C9orf72* HRE-containing plasmid, even though DPR proteins (including poly-GA proteins) were already expressed at these time points. This indicates that the susceptibility to *C9orf72* HRE-derived toxicity might be cell type dependent. In addition, the toxicity of the DPR proteins might be associated with their high molecular weight, aggregated species, which were not detected in the present study in BV-2 cells.

DPR proteins, such as poly-GA, have previously been shown to inhibit proteasomal activity (33, 72, 73). Here, lower levels of polyubiquitinylated proteins were observed in BV-2 cells expressing the *C9orf72* HRE. However, further studies are warranted on the underlying mechanisms of this finding to clarify whether it results from inhibition or enhanced activation of the UPS. Also, it needs to be further elucidated whether this finding in *C9orf72* HRE-expressing BV-2 cells represents a protective mechanism to prevent the TDP-43 and DPR proteins from aggregating. The function of molecular chaperones, such as Hsps, is also intimately linked to the regulation of cellular proteostasis. Members of the HSP70 family have been found to be transcriptionally upregulated in the frontal cortex and cerebellum of *C9orf72* HRE-ALS and *C9orf72* HRE-ALS/FTLD cases as compared with sporadic and control subjects (74, 75). In our study, no changes in Hsp70 levels could be detected, suggesting that the observed upregulation in the human CNS might represent alterations taking place in the later rather than the acute stages of disease. Altogether, our results imply that expression of the *C9orf72* HRE may influence cellular proteostasis through affecting the function of the UPS but not the levels of the Hsps in BV-2 microglial cells.

In previous studies, microglia of *C9orf72* HRE-cases have shown enlarged lysosomes as compared with sporadic ALS cases in *post-mortem* spinal cord at autopsy (75, 76). This suggests that the lysosomal pathway of microglia might be affected by the *C9orf72* HRE. Here, analyses at single cell level indicated that expression of the *C9orf72* HRE caused a slight increase in the LAMP2A levels at 48 h after the transfection. Also, transiently elevated p62 levels at 24 h were detected but these decreased at the 48-h time point. No changes in the levels of these proteins were detected in the WB analyses. These changes, together with the other present data, do not suggest major alterations in the autophagosomal and lysosomal pathways and they were not associated with functional impairment of the BV-2 microglial cells, e.g., in terms of their phagocytic capacity.

In a previous study, the expression of poly-GA in HeLa cells was reported to reduce the translocation of p65, a transcription factor and a subunit of nuclear factor kappa-light-chain-enhancer of activated B cells (NFκB), from the cytoplasm to the nucleus upon TNFα stimulation. Furthermore, it prevented the shuttling of cytoplasmic TDP-43 into the nucleus, indicating that poly-GA impairs nucleocytoplasmic transport mechanisms (64). Here, expression of the *C9orf72* HRE did not alter the response of the BV-2 cells to LPS/IFNγ, as demonstrated by similar levels of NO, IL-6, IL-1β, and TNFα in *C9orf72* HRE-expressing and control cells, suggesting unaltered NFκB signaling and nucleocytoplasmic transport. Furthermore, these results indicate that expression of the *C9orf72* HRE does not lead to impaired activation of the BV-2 cells in response to inflammatory stimuli.

Finally, we hypothesized that the expression of *C9orf72* HRE might lead to a DAM phenotype in BV-2 cells. DAM derive from homeostatic microglia via two stages, the first stage being independent of and the second dependent on Trem2, and each stage is characterized by a specific mRNA profile (58–62). In a previous study, single-cell RNA sequencing from the spinal cords of mSOD1 (G93A) ALS model mice revealed the presence of DAM (59). However, it is not known whether DAM occur and play a role in *C9orf72* HRE-associated ALS or FTLD. In our BV-2 cell model expressing *C9orf72* HRE, there were no alterations in the transcript levels of specific DAM marker genes. We only detected a mild decrease in *Cst7* mRNA levels in *C9orf72* HRE-expressing cells 48 h after transfection. A strong downregulation of *Cst7* expression has previously been reported to lead to increased phagocytic activity of mouse microglia (77). Thus, our finding of only slightly decreased *Cst7* expression is in line with the unaltered phagocytic activity in the BV-2 cells expressing the *C9orf72* HRE. Taken together, acute expression of the *C9orf72* HRE does not appear to lead to dramatic changes in the phenotype or functionality of BV-2 cells.

One limitation of this study is the use of transient transfection. Because of this, it has to be taken into consideration that the untransfected cells might affect the outcome on protein and RNA levels assessed from the total cell lysates. Even though changes in polyubiquitinylated proteins were detectable via WB, moderate changes in TDP-43, p62, and LAMP2A could be observed only using microscopy. This suggests that also other mild changes might be detectable only by single cell–based analyses, especially in model systems using transient transfection. However, so far, no *C9orf72* HRE-expressing stable microglial cell lines are commercially available, and subcloning of the HRE into another plasmid, which encodes a suitable selection marker, has proven to be challenging. Because human microglia of *post-mortem* CNS of *C9orf72* HRE carriers present sense and antisense RNA foci to a much lower extent compared with neurons (17), it could be considered that microglia harbor the *C9orf72* HRE or present HRE-derived pathological hallmarks less frequently than neurons. Indeed, only approximately 13% of microglia showed RNA foci in *post-mortem* brains of *C9orf72* expansion carriers with FTLD (17). Therefore, our transient *in vitro* model with cells exhibiting and not exhibiting the HRE resembles examination of whole brain lysates in bulk. Despite the lack of sense RNA foci, poly-GA and poly-GP DPRs were expressed at high levels in our BV-2 cell model. Interestingly, it has been demonstrated that the DPRs may be secreted and taken up by other cells, indicating cell-to-cell spreading of the DPRs (78, 79). Moreover, a recent study showed that cell-to-cell transmission of poly-GA inhibited proteasome function and led to TDP-43 accumulation in neighboring neurons (73). Therefore, the overexpression of *C9orf72* HRE in transiently transfected BV-2 cells might in fact influence also the adjacent untransfected cells. Another potential limitation of the experiments in the present study is that the BV-2 cells expressing the *C9orf72* HRE were examined in isolation as single cell type cultures. Microglia are known to surveil their surroundings and readily respond to environmental cues. Moreover, it has been shown that microglia isolated from the brain alter their gene expression and thus may present with an altered phenotype as compared with the microglia in their *in vivo* brain environment (80). For example, in human *post-mortem* brains of *C9orf72* HRE carriers, microglia show an activated phenotype and enlarged lysosomes (47, 76, 81). Thus, the lack of a strong functional change in our study might derive from the lack of interactions with other cell types, including neurons. Therefore, in our upcoming studies, we aim to investigate the BV-2 cells harboring the *C9orf72* HRE in co-cultures with neurons and assess whether the neuronal environment alters any of the outcomes investigated in the present study. These studies will also allow investigation of potential non-cell autonomous effects of the microglia. Finally, in general, mouse microglia, especially secondary cell lines, may not fully recapitulate the features of human microglia and, therefore, future investigations in human microglia models are as well needed.

Nevertheless, to our knowledge, we are the first to introduce the *C9orf72* HRE into microglia-like cells and characterize the *C9orf72* HRE-derived pathological hallmarks and their effects on microglial phenotype and functionality. Our data in this acute microglial cell model of *C9orf72* HRE suggest that the *C9orf72* HRE-associated gain-of-toxic-function mechanisms do not affect microglial phenotype or functionality in terms of cell death, phagocytic activity, and inflammatory response despite moderate alterations in the levels of specific proteins involved in these microglial functions. Moreover, acute expression of the *C9orf72* HRE in BV-2 cells does not induce their phenotypic change to DAM-like cells. In the future, induced pluripotent stem cell- or blood monocyte–derived microglial cells from carriers of the *C9orf72* HRE may provide useful model systems to investigate the effects of the *C9orf72* HRE on human microglial cell physiology. In these models, both *C9orf72* HRE-associated loss-of-function (C9orf72 haploinsufficiency) and gain-of-toxic-function mechanisms can be studied concomitantly. As mounting evidence on altered innate immune system and potential glial cell involvement in different neurodegenerative diseases are emerging, it is important to provide insights into their contribution also in the context of *C9orf72* HRE-associated ALS and FTLD pathogenesis, a topic that is currently not well-understood. These investigations may reveal potential new biomarker or therapeutic candidate targets for further translation into clinical settings in the future.

## Data Availability Statement

The raw data supporting the conclusions of this article will be made available by the authors, without undue reservation.

## Author Contributions

AH, HR, MH, ES, MT, TN, and AR conceptualized the design of the study. AB, AA, and JT wrote the TDP-43 analysis software. AH, RW, SL, TN, MT, MH, AA, and JT supervised the study. HR, TH, and PM performed the experiments. HR, TH, SL, and NH validated the methods. HR performed statistical analysis and data presentation. HR and AH wrote the first draft of the article. HR, AH, TH, SL, NH, DH, RW, PM, TN, MT, MH, AB, AA, JT, ES, and AR reviewed and edited the article. All authors contributed to article revision, read, and approved the submitted version.

## Conflict of Interest

The authors declare that the research was conducted in the absence of any commercial or financial relationships that could be construed as a potential conflict of interest.

## Abbreviations

AAV: adeno-associated virus
AF: Alexa Fluor
ALS: amyotrophic lateral sclerosis
BG: background
*Clec7a*: *C-type lectin domain family 7, member a*
CNS: central nervous system
*Cst7*: *cystatin F*
DAPI: 4′,6-diamidino-2-phenylindole
DAM: disease-associated microglia
df: degrees of freedom
DPBS: Dulbecco's phosphate-buffered saline
DPR: dipeptide repeat
FISH: fluorescence *in situ* hybridization
FTLD: frontotemporal lobar degeneration
HEK: human embryonic kidney
HRE: hexanucleotide repeat expansion
Hsp: heat shock protein
ICC: immunocytochemistry
IFN: interferon
IL: interleukin
kDa: kilodalton
LAMP2A: lysosome-associated membrane protein 2 a
LC3B: microtubule associated protein 1 light chain 3 beta
LNA: locked nucleic acid
LPS: lipopolysaccharide
N2a: Neuro-2a
n.d.: not detectable
NFκB: nuclear factor kappa-light-chain-enhancer of activated B cells
NO: nitric oxide
RBP: RNA-binding protein
RT: room temperature
TDP-43: TAR DNA-binding protein 43
TNF: tumor necrosis factor
*Trem2*: *triggering receptor expressed on myeloid cells 2*
*Tyrobp*: *TYRO protein tyrosine kinase binding protein*
UPS: ubiquitin–proteasome system
WB: Western blotting
ZsGreen1: *Zoanthus* sp. green fluorescent protein 1.

## References

[1] Franco R Fernandez-Suarez D. Alternatively activated microglia and macrophages in the central nervous system. Prog Neurobiol. (2015) 131:65–86. 10.1016/j.pneurobio.2015.05.003 26067058

[2] Bachiller S Jimenez-Ferrer I Paulus A Yang Y Swanberg M Deierborg T. Microglia in neurological diseases: a road map to brain-disease dependent-inflammatory response. Front Cell Neurosci. (2018) 12:488. 10.3389/fncel.2018.00488 30618635 PMC6305407

[3] Cooper-Knock J Green C Altschuler G Wei W Bury JJ Heath PR. A data-driven approach links microglia to pathology and prognosis in amyotrophic lateral sclerosis. Acta Neuropathol Commun. (2017) 5:23. 10.1186/s40478-017-0424-x 28302159 PMC5353945

[4] Lee J Hyeon SJ Im H Ryu H Kim Y Ryu H. Astrocytes and microglia as non-cell autonomous players in the pathogenesis of ALS. Exp Neurobiol. (2016) 25:233–40. 10.5607/en.2016.25.5.233 27790057 PMC5081469

[5] Radford RA Morsch M Rayner SL Cole NJ Pountney DL Chung RS. The established and emerging roles of astrocytes and microglia in amyotrophic lateral sclerosis and frontotemporal dementia. Front Cell Neurosci. (2015) 9:414. 10.3389/fncel.2015.00414 26578880 PMC4621294

[6] Onyike CU Diehl-Schmid J. The epidemiology of frontotemporal dementia. Int Rev Psychiatry. (2013) 25:130–7. 10.3109/09540261.2013.776523 23611343 PMC3932112

[7] Van Langenhove T van der Zee J Van Broeckhoven C. The molecular basis of the frontotemporal lobar degeneration-amyotrophic lateral sclerosis spectrum. Ann Med. (2012) 44:817–28. 10.3109/07853890.2012.665471 22420316 PMC3529157

[8] Ling SC Polymenidou M Cleveland DW. Converging mechanisms in ALS and FTD: disrupted RNA and protein homeostasis. Neuron. (2013) 79:416–38. 10.1016/j.neuron.2013.07.033 23931993 PMC4411085

[9] Cooper-Knock J Shaw PJ Kirby J. The widening spectrum of C9ORF72-related disease; genotype/phenotype correlations and potential modifiers of clinical phenotype. Acta Neuropathol. (2014) 127:333–45. 10.1007/s00401-014-1251-9 24493408 PMC3925297

[10] Lillo P Mioshi E Burrell JR Kiernan MC Hodges JR Hornberger M. Grey and white matter changes across the amyotrophic lateral sclerosis-frontotemporal dementia continuum. PLoS ONE. (2012) 7:e43993. 10.1371/journal.pone.0043993 22952843 PMC3430626

[11] DeJesus-Hernandez M Mackenzie IR Boeve BF Boxer AL Baker M Rutherford NJ. Expanded GGGGCC hexanucleotide repeat in noncoding region of C9ORF72 causes chromosome 9p-linked FTD and ALS. Neuron. (2011) 72:245–56. 10.1016/j.neuron.2011.09.011 21944778 PMC3202986

[12] Majounie E Renton AE Mok K Dopper EG Waite A Rollinson S. Frequency of the C9orf72 hexanucleotide repeat expansion in patients with amyotrophic lateral sclerosis and frontotemporal dementia: a cross-sectional study. Lancet Neurol. (2012) 11:323–30. 10.1016/S1474-4422(12)70043-1 22406228 PMC3322422

[13] Renton AE Majounie E Waite A Simon-Sanchez J Rollinson S Gibbs JR. A hexanucleotide repeat expansion in C9ORF72 is the cause of chromosome 9p21-linked ALS-FTD. Neuron. (2011) 72:257–68. 10.1016/j.neuron.2011.09.010 21944779 PMC3200438

[14] Gijselinck I Van Langenhove T van der Zee J Sleegers K Philtjens S Kleinberger G A C9orf72 promoter repeat expansion in a flanders-belgian cohort with disorders of the frontotemporal lobar degeneration-amyotrophic lateral sclerosis spectrum: a gene identification study. Lancet Neurol. (2012) 11:54–65. 10.1016/S1474-4422(11)70261-7 22154785

[15] Saberi S Stauffer JE Jiang J Garcia SD Taylor AE Schulte D. Sense-encoded poly-GR dipeptide repeat proteins correlate to neurodegeneration and uniquely co-localize with TDP-43 in dendrites of repeat-expanded C9orf72 amyotrophic lateral sclerosis. Acta Neuropathol. (2018) 135:459–74. 10.1007/s00401-017-1793-8 29196813 PMC5935138

[16] Gendron TF Bieniek KF Zhang YJ Jansen-West K Ash PE Caulfield T. Antisense transcripts of the expanded C9ORF72 hexanucleotide repeat form nuclear RNA foci and undergo repeat-associated non-ATG translation in c9FTD/ALS. Acta Neuropathol. (2013) 126:829–44. 10.1007/s00401-013-1192-8 24129584 PMC3830741

[17] Mizielinska S Lashley T Norona FE Clayton EL Ridler CE Fratta P. C9orf72 frontotemporal lobar degeneration is characterised by frequent neuronal sense and antisense RNA foci. Acta Neuropathol. (2013) 126:845–57. 10.1007/s00401-013-1200-z 24170096 PMC3830745

[18] Cooper-Knock J Walsh MJ Higginbottom A Robin Highley J Dickman MJ Edbauer D. Sequestration of multiple RNA recognition motif-containing proteins by C9orf72 repeat expansions. Brain. (2014) 137:2040–51. 10.1093/brain/awu120 24866055 PMC4065024

[19] Lagier-Tourenne C Baughn M Rigo F Sun S Liu P Li HR. Targeted degradation of sense and antisense C9orf72 RNA foci as therapy for ALS and frontotemporal degeneration. Proc Natl Acad Sci USA. (2013) 110:E4530–9. 10.1073/pnas.1318835110 24170860 PMC3839752

[20] Sareen D O'Rourke JG Meera P Muhammad AK Grant S Simpkinson M. Targeting RNA foci in iPSC-derived motor neurons from ALS patients with a C9ORF72 repeat expansion. Sci Transl Med. (2013) 5:208ra149. 10.1126/scitranslmed.3007529 24154603 PMC4090945

[21] DeJesus-Hernandez M Finch NA Wang X Gendron TF Bieniek KF Heckman MG. In-depth clinico-pathological examination of RNA foci in a large cohort of C9ORF72 expansion carriers. Acta Neuropathol. (2017) 134:255–69. 10.1007/s00401-017-1725-7 28508101 PMC5508036

[22] Mori K Lammich S Mackenzie IR Forne I Zilow S Kretzschmar H. hnRNP A3 binds to GGGGCC repeats and is a constituent of p62-positive/TDP43-negative inclusions in the hippocampus of patients with C9orf72 mutations. Acta Neuropathol. (2013) 125:413–23. 10.1007/s00401-013-1088-7 23381195

[23] Lee YB Chen HJ Peres JN Gomez-Deza J Attig J Stalekar M. Hexanucleotide repeats in ALS/FTD form length-dependent RNA foci, sequester RNA binding proteins, and are neurotoxic. Cell Rep. (2013) 5:1178–86. 10.1016/j.celrep.2013.10.049 24290757 PMC3898469

[24] Zu T Gibbens B Doty NS Gomes-Pereira M Huguet A Stone MD. Non-ATG-initiated translation directed by microsatellite expansions. Proc Natl Acad Sci USA. (2011) 108:260–5. 10.1073/pnas.1013343108 21173221 PMC3017129

[25] Zu T Liu Y Banez-Coronel M Reid T Pletnikova O Lewis J. RAN proteins and RNA foci from antisense transcripts in C9ORF72 ALS and frontotemporal dementia. Proc Natl Acad Sci USA. (2013) 110:E4968–77. 10.1073/pnas.1315438110 24248382 PMC3870665

[26] Mori K Arzberger T Grasser FA Gijselinck I May S Rentzsch K. Bidirectional transcripts of the expanded C9orf72 hexanucleotide repeat are translated into aggregating dipeptide repeat proteins. Acta Neuropathol. (2013) 126:881–93. 10.1007/s00401-013-1189-3 24132570

[27] Mackenzie IR Arzberger T Kremmer E Troost D Lorenzl S Mori K. Dipeptide repeat protein pathology in C9ORF72 mutation cases: clinico-pathological correlations. Acta Neuropathol. (2013) 126:859–79. 10.1007/s00401-013-1181-y 24096617

[28] Schludi MH May S Grasser FA Rentzsch K Kremmer E Kupper C Distribution of dipeptide repeat proteins in cellular models and C9orf72 mutation cases suggests link to transcriptional silencing. Acta Neuropathol. (2015) 130:537–55. 10.1007/s00401-015-1450-z 26085200 PMC4575390

[29] Ash PE Bieniek KF Gendron TF Caulfield T Lin WL Dejesus-Hernandez M. Unconventional translation of C9ORF72 GGGGCC expansion generates insoluble polypeptides specific to c9FTD/ALS. Neuron. (2013) 77:639–46. 10.1016/j.neuron.2013.02.004 23415312 PMC3593233

[30] May S Hornburg D Schludi MH Arzberger T Rentzsch K Schwenk BM. C9orf72 FTLD/ALS-associated gly-ala dipeptide repeat proteins cause neuronal toxicity and Unc119 sequestration. Acta Neuropathol. (2014) 128:485–503. 10.1007/s00401-014-1329-4 25120191 PMC4159571

[31] Wen X Tan W Westergard T Krishnamurthy K Markandaiah SS Shi Y. Antisense proline-arginine RAN dipeptides linked to C9ORF72-ALS/FTD form toxic nuclear aggregates that initiate *in vitro* and *in vivo* neuronal death. Neuron. (2014) 84:1213–25. 10.1016/j.neuron.2014.12.010 25521377 PMC4632245

[32] Freibaum BD Taylor JP. The role of dipeptide repeats in C9ORF72-related ALS-FTD. Front Mol Neurosci. (2017) 10:35. 10.3389/fnmol.2017.00035 28243191 PMC5303742

[33] Zhang YJ Jansen-West K Xu YF Gendron TF Bieniek KF Lin WL. Aggregation-prone c9FTD/ALS poly(GA) RAN-translated proteins cause neurotoxicity by inducing ER stress. Acta Neuropathol. (2014) 128:505–24. 10.1007/s00401-014-1336-5 25173361 PMC4159567

[34] Kwon I Xiang S Kato M Wu L Theodoropoulos P Wang T. Poly-dipeptides encoded by the C9orf72 repeats bind nucleoli, impede RNA biogenesis, and kill cells. Science. (2014) 345:1139–45. 10.1126/science.1254917 25081482 PMC4459787

[35] Mahoney CJ Beck J Rohrer JD Lashley T Mok K Shakespeare T. Frontotemporal dementia with the C9ORF72 hexanucleotide repeat expansion: clinical, neuroanatomical and neuropathological features. Brain. (2012) 135:736–50. 10.1093/brain/awr361 22366791 PMC3286330

[36] Arai T Hasegawa M Akiyama H Ikeda K Nonaka T Mori H. TDP-43 is a component of ubiquitin-positive tau-negative inclusions in frontotemporal lobar degeneration and amyotrophic lateral sclerosis. Biochem Biophys Res Commun. (2006) 351:602–11. 10.1016/j.bbrc.2006.10.093 17084815

[37] Dedeene L Van Schoor E Race V Moisse M Vandenberghe R Poesen K. An ALS case with 38 (G4C2)-repeats in the C9orf72 gene shows TDP-43 and sparse dipeptide repeat protein pathology. Acta Neuropathol. (2019) 137:855–8. 10.1007/s00401-019-01996-z 30919029

[38] Shinagawa S Naasan G Karydas AM Coppola G Pribadi M Seeley WW. Clinicopathological study of patients with C9ORF72-associated frontotemporal dementia presenting with delusions. J Geriatr Psychiatry Neurol. (2015) 28:99–107. 10.1177/0891988714554710 25342578 PMC4408221

[39] Lee SM Asress S Hales CM Gearing M Vizcarra JC Fournier CN. TDP-43 cytoplasmic inclusion formation is disrupted in C9orf72-associated amyotrophic lateral sclerosis/frontotemporal lobar degeneration. Brain Commun. (2019) 1:fcz014. 10.1093/braincomms/fcz014 31633109 PMC6788139

[40] Al-Sarraj S King A Troakes C Smith B Maekawa S Bodi I. p62 positive, TDP-43 negative, neuronal cytoplasmic and intranuclear inclusions in the cerebellum and hippocampus define the pathology of C9orf72-linked FTLD and MND/ALS. Acta Neuropathol. (2011) 122:691–702. 10.1007/s00401-011-0911-2 22101323

[41] Chou CC Zhang Y Umoh ME Vaughan SW Lorenzini I Liu F. TDP-43 pathology disrupts nuclear pore complexes and nucleocytoplasmic transport in ALS/FTD. Nat Neurosci. (2018) 21:228–39. 10.1038/s41593-017-0047-3 29311743 PMC5800968

[42] Simon-Sanchez J Dopper EG Cohn-Hokke PE Hukema RK Nicolaou N Seelaar H. The clinical and pathological phenotype of C9ORF72 hexanucleotide repeat expansions. Brain. (2012) 135:723–35. 10.1093/brain/awr353 22300876

[43] Blasi E Barluzzi R Bocchini V Mazzolla R Bistoni F. Immortalization of murine microglial cells by a v-raf/v-myc carrying retrovirus. J Neuroimmunol. (1990) 27:229–37. 10.1016/0165-5728(90)90073-V 2110186

[44] Chew J Gendron TF Prudencio M Sasaguri H Zhang YJ Castanedes-Casey M. Neurodegeneration. C9ORF72 repeat expansions in mice cause TDP-43 pathology, neuronal loss, and behavioral deficits. Science. (2015) 348:1151–4. 10.1126/science.aaa9344 25977373 PMC4692360

[45] Nicholson AM Zhou X Perkerson RB Parsons TM Chew J Brooks M. Loss of tmem106b is unable to ameliorate frontotemporal dementia-like phenotypes in an AAV mouse model of C9ORF72-repeat induced toxicity. Acta Neuropathol Commun. (2018) 6:42. 10.1186/s40478-018-0545-x 29855382 PMC5984311

[46] Budini M Buratti E Morselli E Criollo A. Autophagy and its impact on neurodegenerative diseases: new roles for TDP-43 and C9orf72. Front Mol Neurosci. (2017) 10:170. 10.3389/fnmol.2017.00170 28611593 PMC5447761

[47] Cooper-Knock J Hewitt C Highley JR Brockington A Milano A Man S. Clinico-pathological features in amyotrophic lateral sclerosis with expansions in C9ORF72. Brain. (2012) 135:751–64. 10.1093/brain/awr365 22366792 PMC3286332

[48] Schipper LJ Raaphorst J Aronica E Baas F de Haan R de Visser M. Prevalence of brain and spinal cord inclusions, including dipeptide repeat proteins, in patients with the C9ORF72 hexanucleotide repeat expansion: a systematic neuropathological review. Neuropathol Appl Neurobiol. (2016) 42:547–60. 10.1111/nan.12284 26373655

[49] Nishihira Y Tan CF Onodera O Toyoshima Y Yamada M Morita T. Sporadic amyotrophic lateral sclerosis: two pathological patterns shown by analysis of distribution of TDP-43-immunoreactive neuronal and glial cytoplasmic inclusions. Acta Neuropathol. (2008) 116:169–82. 10.1007/s00401-008-0385-z 18481073

[50] Kalmar B Greensmith L. Cellular chaperones as therapeutic targets in ALS to restore protein homeostasis and improve cellular function. Front Mol Neurosci. (2017) 10:251. 10.3389/fnmol.2017.00251 28943839 PMC5596081

[51] D'Arcy MS. Cell death: a review of the major forms of apoptosis, necrosis and autophagy. Cell Biol Int. (2019) 43:582–92. 10.1002/cbin.11137 30958602

[52] Vernon PJ Tang D. Eat-me: autophagy, phagocytosis, and reactive oxygen species signaling. Antioxid Redox Signal. (2013) 18:677–91. 10.1089/ars.2012.4810 22871044 PMC3632094

[53] Webster CP Smith EF Bauer CS Moller A Hautbergue GM Ferraiuolo L. The C9orf72 protein interacts with Rab1a and the ULK1 complex to regulate initiation of autophagy. EMBO J. (2016) 35:1656–76. 10.15252/embj.201694401 27334615 PMC4969571

[54] Leskela S Huber N Rostalski H Natunen T Remes AM Takalo M. C9orf72 Proteins regulate autophagy and undergo autophagosomal or proteasomal degradation in a cell type-dependent manner. Cells. (2019) 8:1233. 10.3390/cells8101233 31658762 PMC6829620

[55] Merrill JE Ignarro LJ Sherman MP Melinek J Lane TE. Microglial cell cytotoxicity of oligodendrocytes is mediated through nitric oxide. J Immunol. (1993) 151:2132–41. 8102159

[56] Chao CC Hu S Molitor TW Shaskan EG Peterson PK. Activated microglia mediate neuronal cell injury via a nitric oxide mechanism. J Immunol. (1992) 149:2736–41. 1383325

[57] Olson JK Miller SD. Microglia initiate central nervous system innate and adaptive immune responses through multiple TLRs. J Immunol. (2004) 173:3916–24. 10.4049/jimmunol.173.6.3916 15356140

[58] Deczkowska A Keren-Shaul H Weiner A Colonna M Schwartz M Amit I. Disease-associated microglia: a universal immune sensor of neurodegeneration. Cell. (2018) 173:1073–81. 10.1016/j.cell.2018.05.003 29775591

[59] Keren-Shaul H Spinrad A Weiner A Matcovitch-Natan O Dvir-Szternfeld R Ulland TK. A unique microglia type associated with restricting development of Alzheimer's disease. Cell. (2017) 169:1276–90.e17. 10.1016/j.cell.2017.05.018 28602351

[60] Krasemann S Madore C Cialic R Baufeld C Calcagno N El Fatimy R. The TREM2-APOE pathway drives the transcriptional phenotype of dysfunctional microglia in neurodegenerative diseases. Immunity. (2017) 47:566–81.e9. 10.1016/j.immuni.2017.08.008 28930663 PMC5719893

[61] Rangaraju S Dammer EB Raza SA Rathakrishnan P Xiao H Gao T. Identification and therapeutic modulation of a pro-inflammatory subset of disease-associated-microglia in Alzheimer's disease. Mol Neurodegener. (2018) 13:24. 10.1186/s13024-018-0254-8 29784049 PMC5963076

[62] Spiller KJ Restrepo CR Khan T Dominique MA Fang TC Canter RG. Microglia-mediated recovery from ALS-relevant motor neuron degeneration in a mouse model of TDP-43 proteinopathy. Nat Neurosci. (2018) 21:329–40. 10.1038/s41593-018-0083-7 29463850 PMC5857237

[63] Lee YB Baskaran P Gomez-Deza J Chen HJ Nishimura AL Smith BN C9orf72 poly GA RAN-translated protein plays a key role in amyotrophic lateral sclerosis via aggregation and toxicity. Hum Mol Genet. (2017) 26:4765–77. 10.1093/hmg/ddx350 28973350 PMC5886201

[64] Khosravi B Hartmann H May S Mohl C Ederle H Michaelsen M. Cytoplasmic poly-GA aggregates impair nuclear import of TDP-43 in C9orf72 ALS/FTLD. Hum Mol Genet. (2017) 26:790–800. 10.1093/hmg/ddw432 28040728 PMC5409121

[65] McGoldrick P Zhang M van Blitterswijk M Sato C Moreno D Xiao S. Unaffected mosaic C9orf72 case: RNA foci, dipeptide proteins, but upregulated C9orf72 expression. Neurology. (2018) 90:e323–31. 10.1212/WNL.0000000000004865 29282338 PMC5798652

[66] Schludi MH Becker L Garrett L Gendron TF Zhou Q Schreiber F. Spinal poly-GA inclusions in a C9orf72 mouse model trigger motor deficits and inflammation without neuron loss. Acta Neuropathol. (2017) 134:241–54. 10.1007/s00401-017-1711-0 28409281 PMC5508040

[67] Nonaka T Masuda-Suzukake M Hosokawa M Shimozawa A Hirai S Okado H. C9ORF72 dipeptide repeat poly-GA inclusions promote intracellular aggregation of phosphorylated TDP-43. Hum Mol Genet. (2018) 27:2658–70. 10.1093/hmg/ddy174 29750243

[68] Liu Y Pattamatta A Zu T Reid T Bardhi O Borchelt DR. C9orf72 BAC mouse model with motor deficits and neurodegenerative features of ALS/FTD. Neuron. (2016) 90:521–34. 10.1016/j.neuron.2016.04.005 27112499

[69] Solomon DA Stepto A Au WH Adachi Y Diaper DC Hall R. A feedback loop between dipeptide-repeat protein, TDP-43 and karyopherin-alpha mediates C9orf72-related neurodegeneration. Brain. (2018) 141:2908–24. 10.1093/brain/awy241 30239641 PMC6158706

[70] Dafinca R Scaber J Ababneh N Lalic T Weir G Christian H. C9orf72 Hexanucleotide expansions are associated with altered endoplasmic reticulum calcium homeostasis and stress granule formation in induced pluripotent stem cell-derived neurons from patients with amyotrophic lateral sclerosis and frontotemporal dementia. Stem Cells. (2016) 34:2063–78. 10.1002/stem.2388 27097283 PMC4979662

[71] Onesto E Colombrita C Gumina V Borghi MO Dusi S Doretti A. Gene-specific mitochondria dysfunctions in human TARDBP and C9ORF72 fibroblasts. Acta Neuropathol Commun. (2016) 4:47. 10.1186/s40478-016-0316-5 27151080 PMC4858818

[72] Gupta R Lan M Mojsilovic-Petrovic J Choi WH Safren N Barmada S. The proline/arginine dipeptide from hexanucleotide repeat expanded C9ORF72 inhibits the proteasome. eNeuro. (2017) 4:ENEURO.0249-16.2017. 10.1523/ENEURO.0249-16.2017 28197542 PMC5282547

[73] Khosravi B LaClair KD Riemenschneider H Zhou Q Frottin F Mareljic N. Cell-to-cell transmission of C9orf72 poly-(Gly-Ala) triggers key features of ALS/FTD. EMBO J. (2020) 39:e102811. 10.15252/embj.2019102811 32175624 PMC7156967

[74] Prudencio M Belzil VV Batra R Ross CA Gendron TF Pregent LJ. Distinct brain transcriptome profiles in C9orf72-associated and sporadic ALS. Nat Neurosci. (2015) 18:1175–82. 10.1038/nn.4065 26192745 PMC4830686

[75] Mordes DA Prudencio M Goodman LD Klim JR Moccia R Limone F. Dipeptide repeat proteins activate a heat shock response found in C9ORF72-ALS/FTLD patients. Acta Neuropathol Commun. (2018) 6:55. 10.1186/s40478-018-0555-8 29973287 PMC6031111

[76] O'Rourke JG Bogdanik L Yanez A Lall D Wolf AJ Muhammad AK. C9orf72 is required for proper macrophage and microglial function in mice. Science. (2016) 351:1324–9. 10.1126/science.aaf1064 26989253 PMC5120541

[77] Kang SS Ebbert MTW Baker KE Cook C Wang X Sens JP. Microglial translational profiling reveals a convergent APOE pathway from aging, amyloid, and tau. J Exp Med. (2018) 215:2235–45. 10.1084/jem.20180653 30082275 PMC6122978

[78] Westergard T Jensen BK Wen X Cai J Kropf E Iacovitti L. Cell-to-cell transmission of dipeptide repeat proteins linked to C9orf72-ALS/FTD. Cell Rep. (2016) 17:645–52. 10.1016/j.celrep.2016.09.032 27732842 PMC5078984

[79] Zhou Q Lehmer C Michaelsen M Mori K Alterauge D Baumjohann D. Antibodies inhibit transmission and aggregation of C9orf72 poly-GA dipeptide repeat proteins. EMBO Mol Med. (2017) 9:687–702. 10.15252/emmm.201607054 28351931 PMC5412769

[80] Hasselmann J Coburn MA England W Figueroa Velez DX Kiani Shabestari S Tu CH. Development of a chimeric model to study and manipulate human microglia *in vivo*. Neuron. (2019) 103:1016–33.e10. 10.1016/j.neuron.2019.07.002 31375314 PMC7138101

[81] Brettschneider J Toledo JB Van Deerlin VM Elman L McCluskey L Lee VM. Microglial activation correlates with disease progression and upper motor neuron clinical symptoms in amyotrophic lateral sclerosis. PLoS ONE. (2012) 7:e39216. 10.1371/journal.pone.0039216 22720079 PMC3375234

