# Supplementary material for: BV-2 Microglial Cells Overexpressing C9orf72 Hexanucleotide Repeat Expansion Produce DPR Proteins and Show Normal Functionality but No RNA Foci
Source: Front Neurol. 2020 Oct 6;11:550140. doi: 10.3389/fneur.2020.550140 (PMC7573144; doi:10.3389/fneur.2020.550140)
Supplement: Supplementary file 1 [file Data_Sheet_1.docx]

Supplementary Material


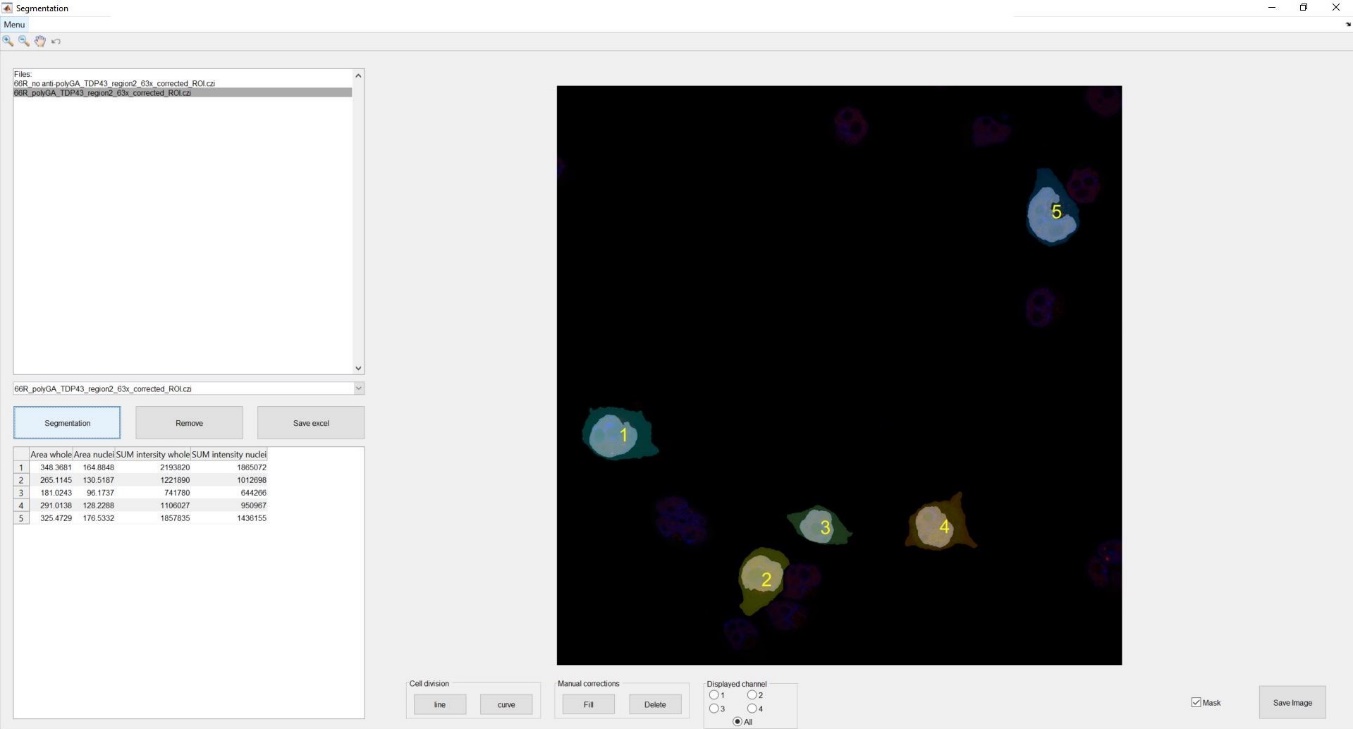


**Supplementary Figure 1.** Exemplary image of the segmentation toolbox used for TDP-43 translocation analysis. Cell bodies and corresponding nuclei of ZsGreen1-positive cells are annotated and numbered. SUM intensities for TDP-43 signal as well as area size of nuclei and whole cell body respectively are calculated for further analysis.


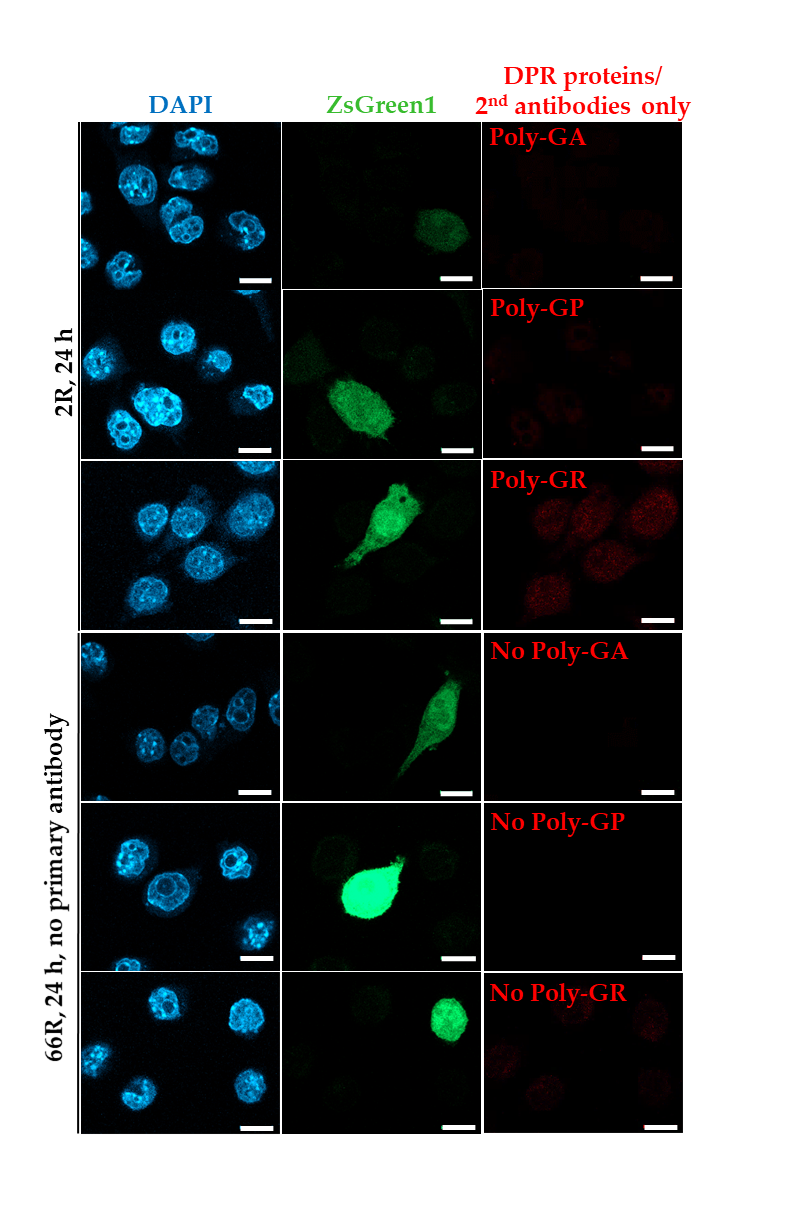


**Supplementary Figure 2**. Validation of antibody specificity towards DPR proteins. Representative images 24 h after transfection of BV-2 cells co-transfected with 2R or 66R in combination with ZsGreen1 encoding plasmids. Representative images for two independent cell culture experiments. Scale bar = 10 µm


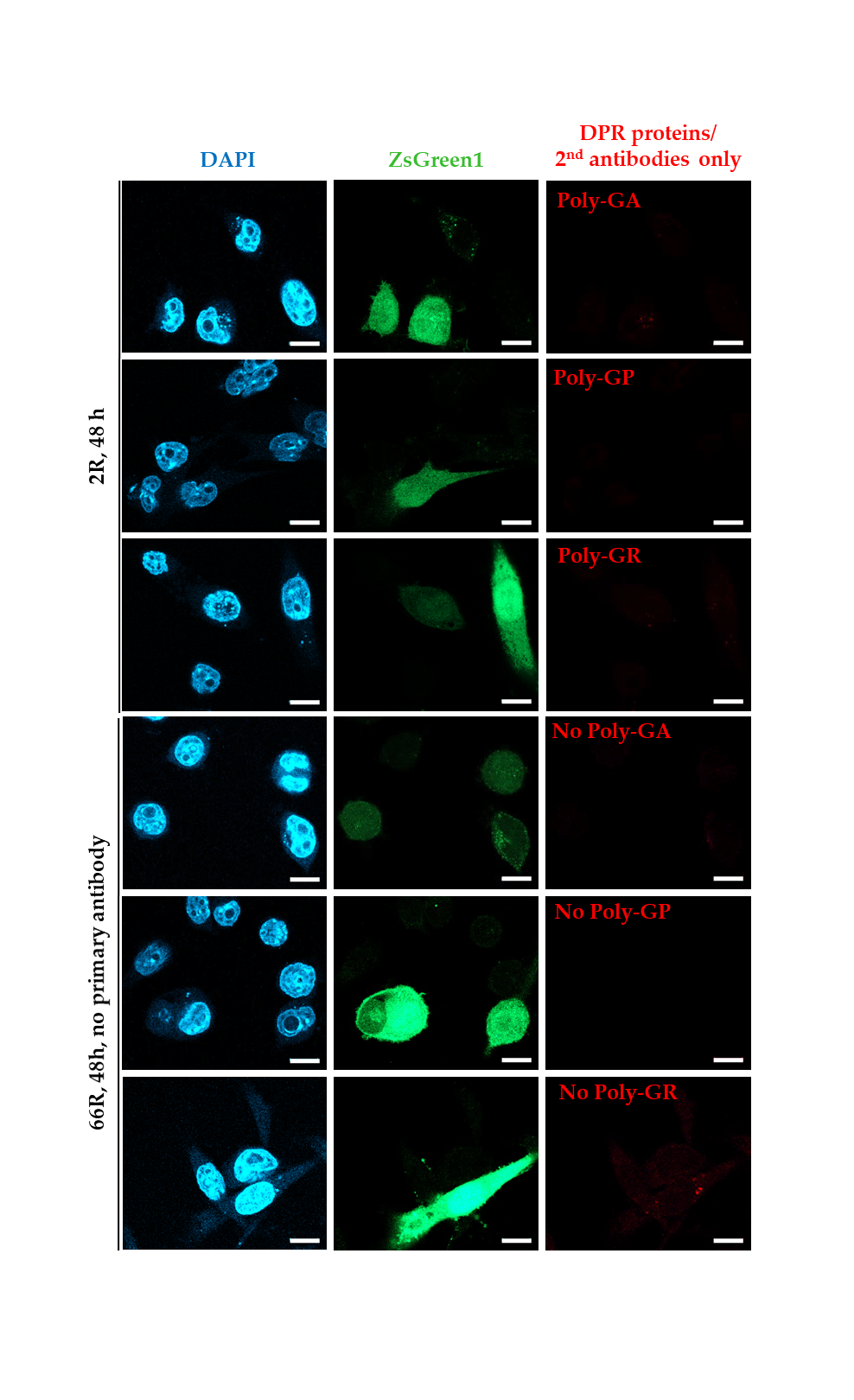


**Supplementary Figure 3.** Validation of antibody specificity towards DPR proteins. Representative images 48 h after transfection of BV-2 cells co-transfected with 2R or 66R and ZsGreen1 encoding plasmids. Representative images for three independent transfections. Scale bar = 10 µm


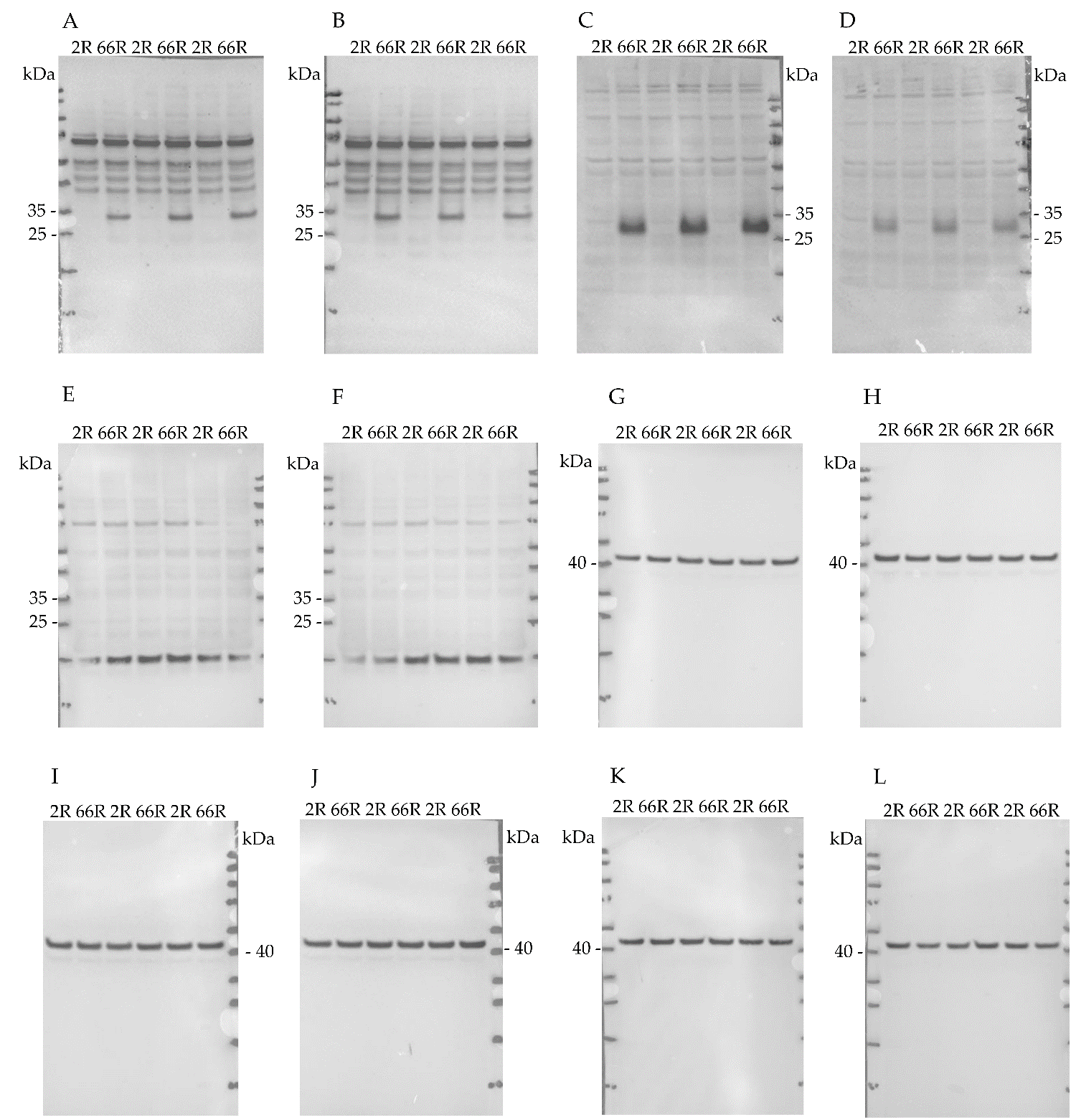


**Supplementary Figure 4.** Whole Western Blot images for poly-GA (**A**, **B**), poly-GP (**C**, **D**), poly-GR (**E**, **F**), and corresponding β-actin (**G**, **H**: poly-GA blots; **I, J**: poly-GP blots; **K, L**: poly-GR blots) signals shown as cropped images in Figure 2. BV-2 cells were transiently transfected with 2R or 66R plasmids. Protein samples were harvested 24 h (**A**, **C**, **E**, **G**, **I**, **K**) or 48 h (**B**, **D**, **F**, **H**, **J**, **L**) after transfection. Representative blots of four independent experiments are shown.


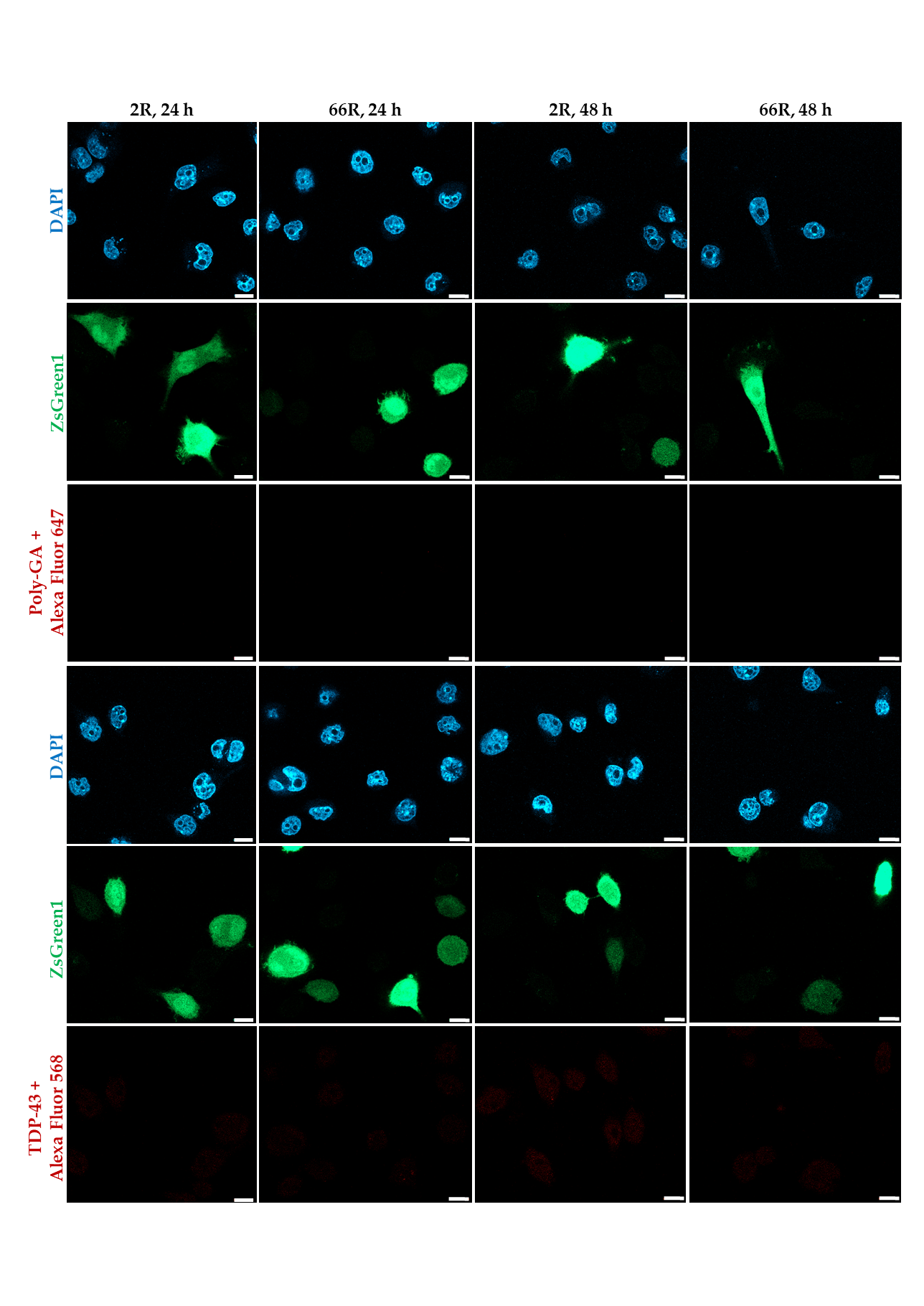


**Supplementary Figure 5.** Validation of low cross-reactivity of secondary antibodies in co-transfected BV-2 cells double stained for TDP-43 and poly-GA. BV-2 cells transfected with either 2R or 66R plasmids in combination with a plasmid coding for ZsGreen1 (green) show no cross-reactivity of anti-poly-GA with Alexa Fluor^®^ 647 (red) (**A**) or anti-TDP-43 with Alexa Fluor^®^ 568 (red) (**B**) 24 h or 48 h after transfection. Representative images of three independent experiments. Nuclei were stained with DAPI (blue). Scale bar = 10 µm. Abbrev.: DAPI = 4′,6-diamidino-2-phenylindole, TDP-43 = TAR DNA-binding protein 43, ZsGreen1 = *Zoanthus* sp. green fluorescent protein


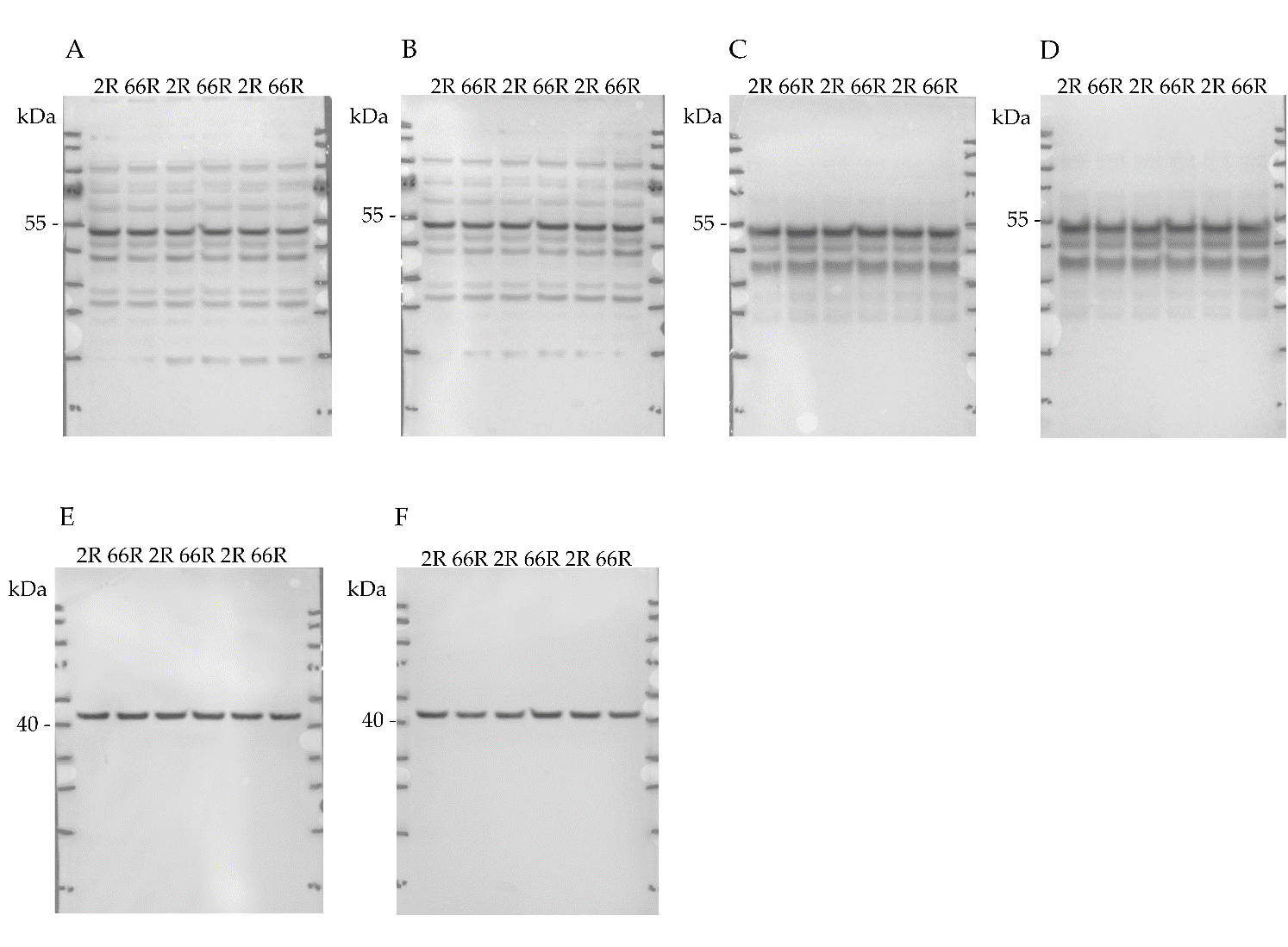


**Supplementary Figure 6.** Whole Western Blot images for phospho-TDP-43 (**A**, **B**), TDP-43 (**C**, **D**), and β-actin (**E**, **F**) signals shown as cropped images in Figure 3. BV-2 cells were transiently transfected with 2R or 66R plasmids. Protein samples were harvested 24 h (**A**, **C**, **E**) or 48 h (**B**, **D**, **F**) after transfection. Representative blots of four independent experiments are shown.


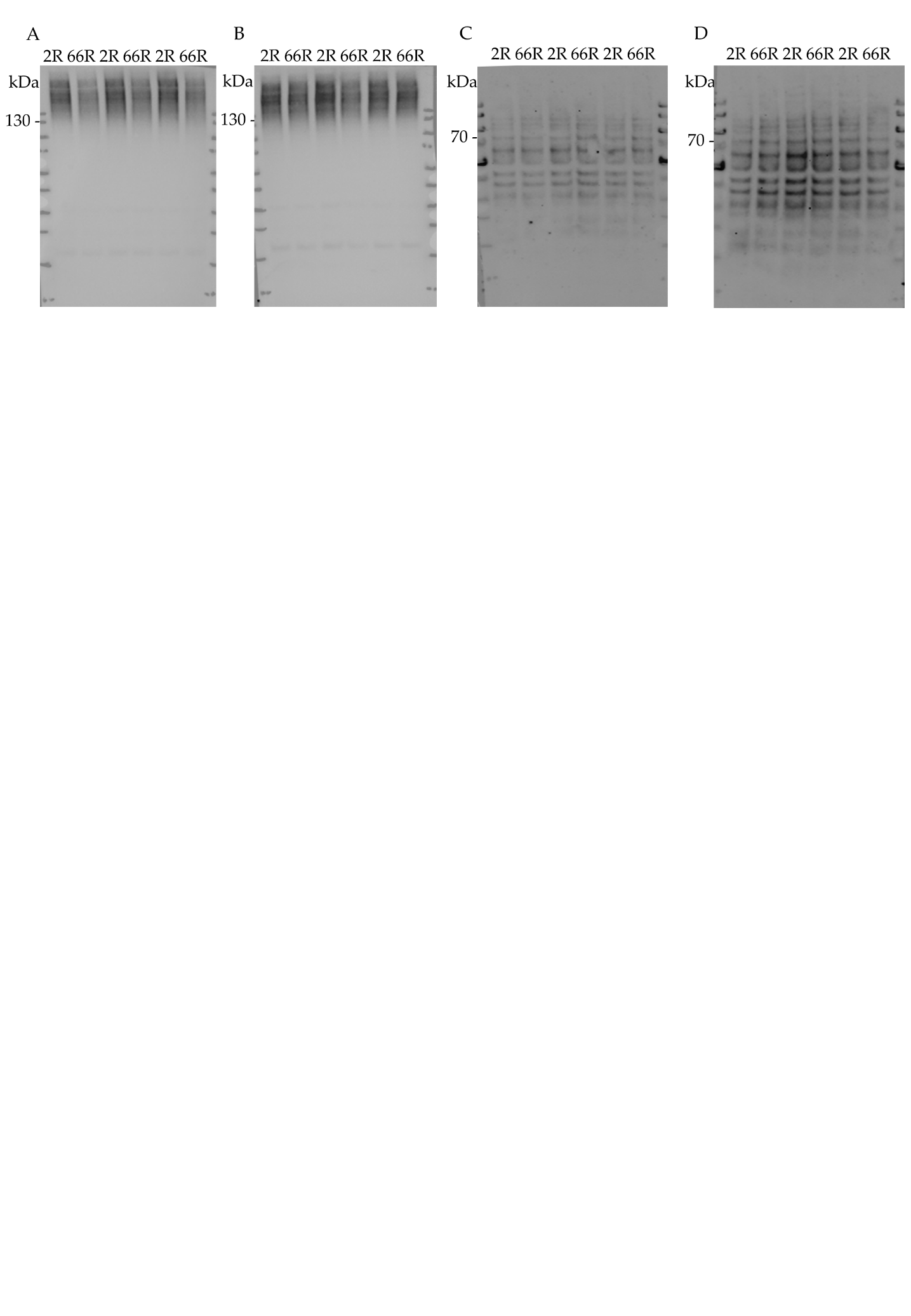


**Supplementary Figure 7.** Whole Western Blot images for polyubiquitinylated proteins (**A**, **B**) and Hsp70 (**C**, **D**) shown as cropped images in Figure 5. BV-2 cells were transiently transfected with 2R or 66R plasmids. Protein samples were harvested 24 h (**A**, **C**) or 48 h (**B**, **D**) after transfection. Representative blots of four independent experiments are shown.


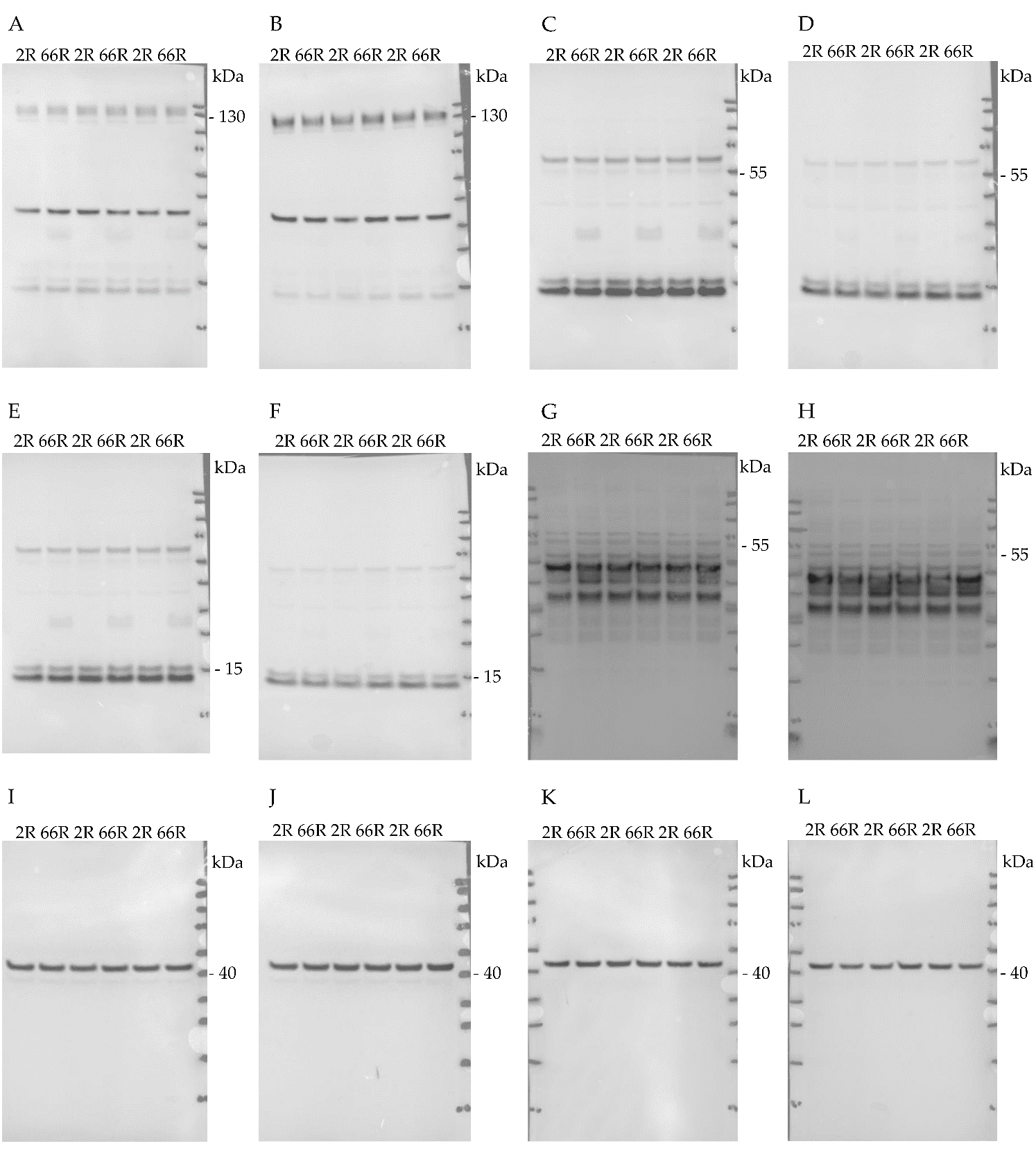


**Supplementary Figure 8.** Whole Western Blot images for LAMP-2A (**A**, **B**), p62(**C**, **D**), LC3B (**E**, **F**), C9orf72 (**G**, **H**), and corresponding β-actin (**I**, **J**: LAMP-2A, p62 and LC3B blot; **K, L**: C9orf72 blot) signals shown as cropped images in Figure 7. BV-2 cells were transiently transfected with 2R or 66R plasmids. Protein samples were harvested 24 h (**A**, **C**, **E, G, I, K**) or 48 h (**B**, **D**, **F, H, J, L**) after transfection. Representative blots of four independent experiments are shown.

**Supplementary Table 1.** Information on primers used for RT-qPCR

|  | Primer sequence (5’ 🡪 3’) | |  |
| --- | --- | --- | --- |
| Gene name | Forward | Reverse | Annealing temp. [°C] |
| *Actb* | GGCTGTATTCCCCTCCATCG | CCAGTTGGTAACAATGCCATGT | 58 |
| *Clec7a* | AACCACAAGCCCACAGAATCA | CGGTGAGACGATGTTTGGCT | 58 |
| *Cst7* | GTGAAGCCAGGATTCCCCAA | AACAGGCCTCAGCAGAATCG | 55 |
| *Trem2* | TGGAACCGTCACCATCACTC | TGGTCATCTAGAGGGTCCTCC | 55 |
| *Tyrobp* | ACCCGGAAACAACACATTGC | TTGCCTCTGTGTGTTGAGGT | 55 |
